# The hippocampal subgranular zone neurogenic niche: a modular perspective on neural stem cell regulation

**DOI:** 10.3389/fncel.2026.1887577

**Published:** 2026-08-18

**Authors:** Tengyu Zhao, Yuhan Zhou, Pengyu Pan, Ying Jia, Xinyue Zhang, Zeyuan An, Jingdong Yan, Quan Li, Yanyan Zhou

**Affiliations:** 1 School of Basic Medicine, Heilongjiang University of Chinese Medicine, Harbin, China; 2 The Second Affiliated Hospital of Heilongjiang University of Chinese Medicine, Harbin, China; 3 The First Affiliated Hospital of Heilongjiang University of Chinese Medicine, Harbin, China; 4 Key Laboratory of Basic Theory Research on Traditional Chinese Medicine, Harbin, China

**Keywords:** aging, Alzheimer’s disease, microenvironment, neural stem cells, neurogenesis, neurogenic niche, subgranular zone

## Abstract

The subgranular zone (SGZ) of the hippocampus represents a principal site of adult neurogenesis and exhibits distinct structural and organizational features. Increasing evidence indicates that neural stem cell (NSC) behavior in the SGZ is not solely determined by intrinsic cellular properties, but is critically shaped by its surrounding neurogenic niche. However, current studies largely describe niche-derived regulatory factors in isolation, and a systematic framework integrating these diverse signals remains lacking. In this review, we propose a structured perspective that links the unique anatomical and organizational characteristics of the SGZ to its regulatory mechanisms. We conceptualize SGZ niche regulation as a multi-dimensional and non-hierarchical system, in which multiple interacting components—including spatial organization, metabolic–vascular support, neural circuit activity, immune modulation, and extracellular matrix–mediated signaling—collectively govern NSC state transitions. Within this framework, neurogenesis is understood as an emergent outcome of coordinated changes across these regulatory dimensions rather than the result of single-factor control. We further discuss how shifts in the neurogenic niche under pathological conditions—such as aging, Alzheimer’s disease, and chronic cerebral hypoperfusion (CCH) —reshape NSC behavior, driving maladaptive responses that may ultimately lead to dysregulated neurogenesis. By providing a modular and system-level perspective, this review offers a conceptual basis for understanding SGZ regulation and may help identify potential targets for restoring neurogenic capacity.

## 1 Introduction

Since the 1960s, when Joseph Altman and his colleagues first reported the generation of new neurons in adult rodents within the subventricular zone (SVZ) of the lateral ventricles and the subgranular zone (SGZ) of the dentate gyrus (DG), the plasticity and regenerative potential of the mammalian central nervous system have been systematically re-examined (Altman, 1962; Altman and Das, 1965). Accumulating evidence now suggests that the human brain may sustain neurogenesis throughout adult life through neural stem cells (NSCs), although the extent of this process remains incompletely understood (Knoth et al., 2010; Boldrini et al., 2018; Spalding et al., 2013; Moreno-Jiménez et al., 2021). Although the neurogenic capacity of NSCs gradually declines with age (Aran et al., 2025), studies indicate that during human aging, alterations in the neural stem cell microenvironment and the marked increase in the proportion of quiescent stem cells may exert a more critical influence than changes in stem cell number itself (Boldrini et al., 2018). These findings suggest that the regulation of adult neurogenesis does not rely solely on the intrinsic properties of stem cells, but instead depends profoundly on the organizational features of the niche in which they reside and on mechanisms governing transitions between cellular states.

Among the two principal regions of adult neurogenesis, NSCs in the SGZ generate glutamatergic granule neurons locally and represent a major source of hippocampal neurogenesis, thereby contributing to learning, memory, and emotional regulation (Miller and Sahay, 2019; Gonçalves et al., 2016). In the SVZ, neural progenitor cells undergo long-distance migration to the olfactory bulb (Elliott et al., 2025). By contrast, the entire SGZ neurogenic sequence—from quiescence and activation to differentiation and maturation—occurs locally within the deep dentate gyrus, without long-range migration (Liang et al., 2025). This spatial confinement confers a stronger dependence on the surrounding niche environment. The fate determination of NSCs in the SGZ is therefore not an isolated cell-autonomous process but instead reflects an integrated response, within specific temporal windows, to signals derived from multiple sources, including the vasculature, glial cells, neural circuits, and the extracellular matrix (ECM) (Liang et al., 2025; Ma et al., 2025). Together, these feedback signals determine whether NSCs remain quiescent, enter activation, undergo lineage differentiation, or return to a steady state, thereby shaping the overall output of adult neurogenesis.

At the same time, the multiple progenitor origins of NSCs and their diverse differentiation potentials confer pronounced structural heterogeneity across distinct stem cell pools (Edri et al., 2015). The SVZ and SGZ differ markedly in their spatial organization and in the ways they interface with the external environment, giving rise to fundamentally distinct regulatory logics. Recent studies have revealed a close coupling among regional characteristics within the SVZ, niche architecture, and the physiological state of the organism, indicating a form of state-dependent regulation that is closely linked to spatial organization (Chaker et al., 2024). In contrast, the intrinsic relationship between the structural organization of the SGZ and its modes of state regulation has not yet been systematically integrated.

Accordingly, this review first outlines the structural features of the NSC niche in the SGZ and interprets the origins of its functional heterogeneity from a niche-based perspective. It then proposes a modular framework for niche regulation organized along five dimensions: spatial anchoring, metabolic permission, neural circuit feedback, maintenance of immune homeostasis, and interfaces for signal integration. Finally, we discuss how this multidimensional niche regulatory network may become shifted under conditions such as aging, Alzheimer’s disease, CCH, and ischemic stroke, and consider the potential consequences of such alterations. Through this integrative perspective, we aim to provide a structured explanation of how SGZ neurogenesis may transition from adaptive regulation to maladaptive remodeling within its niche context.

## 2 Understanding functional heterogeneity from the structural organization of the SGZ niche

**Figure 1:**
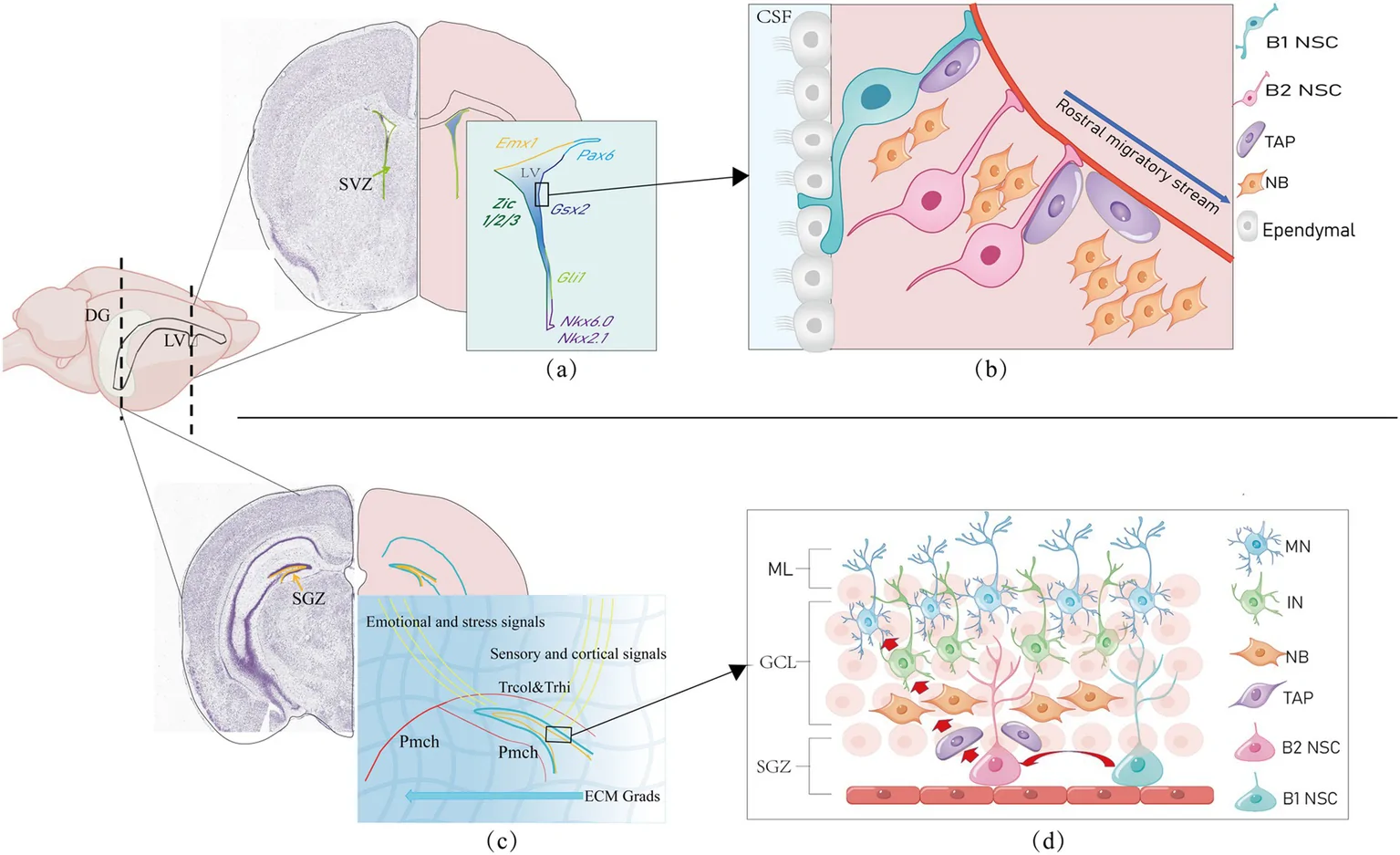
Structural organization of the neurogenic niche and differentiation patterns of adult neurogenesis in the SVZ and SGZ. The subventricular zone (SVZ) and subgranular zone (SGZ) differ markedly in the structural organization of their neurogenic niches, their interactions with the surrounding environment, and the manifestation of stem cell heterogeneity. The SVZ exhibits a spatially regionalized niche architecture with defined transcriptional domains and long-distance neuronal migration, whereas the SGZ lacks stable adult spatial compartmentalization and instead operates as a locally embedded niche characterized by gradient-like heterogeneity. These distinctions provide the structural basis for the divergent regulatory logics of niche organization and adult neurogenesis in the two regions. **(a)** Schematic illustration of the anatomical location of the SVZ along the lateral ventricles, together with region-specific transcription factor expression patterns (e.g., Emx1, Pax6, Gsx2, Nkx2.1, and Zic1/2/3), highlighting the spatial regionalization of SVZ NSCs populations. **(b)** Cellular organization of the SVZ niche, including B1 and B2 NSCs, transit-amplifying progenitor (TAP), neuroblasts (NBs), and ependymal cells, illustrating the structural basis for contact-dependent signaling and multi-source input integration. **(c)** Schematic representation of the anatomical location of the SGZ within the dentate gyrus (DG), together with longitudinal gradients of functional inputs, including sensory/cortical and emotional/stress-related signals, as well as ECM-related gradients. **(d)** Schematic illustration of the neurogenic process in the SGZ, showing the progression from quiescent NSCs to activated progenitors, neuroblasts, and mature neurons within a locally confined niche. This spatial confinement is associated with strong coupling among niche components, resulting in tightly integrated and context-dependent regulation of neurogenesis. B1 NSC, type B1 neural stem cell; B2 NSC, type B2 neural stem cell; CSF, cerebrospinal fluid; DG, dentate gyrus; ECM grads, extracellular matrix gradients; GCL, granule cell layer; IN, immature neuron; LV, lateral ventricle; LW, Lateral Wall; ML, molecular layer; MN, mature neuron; NB, neuroblast; SGZ, subgranular zone; TAP, transit amplifying progenitor. Mouse brain histological diagram. Reproduced from the Allen Mouse Brain Reference Atlas (Version 3, 2011), Allen Institute for Brain Science (https://mouse.brain-map.org/static/atlas). © 2011 Allen Institute for Brain Science. Reproduced with permission.

Heterogeneity within neural stem cell pools represents a fundamental feature of adult neurogenesis and reflects differences in cellular states among distinct subpopulations within the stem cell compartment (Chaker et al., 2024). Such variation arises from the cumulative interplay among intrinsic properties of specific subpopulations, their developmental or differentiation stages, and regulatory influences from the surrounding microenvironment (niche) (Chaker et al., 2024; Dimitrakopoulos et al., 2022). These interactions ultimately manifest as observable phenotypic differences, reflecting the intrinsic diversity of stem cells in terms of fate decisions, regulatory mechanisms, and transient cellular states. Notably, the neural stem cell pools of SGZ and SVZ exhibit pronounced differences in how this heterogeneity is organized and expressed (Figure 1).

In the SVZ, NSCs display a relatively well-defined pattern of spatial regionalization. Lineage-tracing studies have shown that this regional heterogeneity can be traced back to progenitor cells originating from distinct embryonic germinal zones, and that relatively stable spatial patterns of transcription factor expression are retained into adulthood, including dorsal Emx1, ventral Nkx2.1, lateral Gsx2, and medial Zic1/2/3 (López-Juárez et al., 2013; Delgado and Lim, 2015; Mizrak et al., 2019; Kohwi et al., 2007). In addition, NSCs in the SVZ extend apical processes that contact the cerebrospinal fluid, while their basal processes connect with the vascular network, thereby enabling the spatial integration of multiple signaling inputs derived from the cerebrospinal fluid, the circulatory system, and local cellular populations (Fuentealba et al., 2012; Fame et al., 2023; Lim and Alvarez-Buylla, 2016). This well-defined structural regionalization and pattern of signal accessibility confer a largely region-dependent mode of regulation on SVZ neurogenesis (Chaker et al., 2024).

In contrast, current evidence has not demonstrated the presence of stable lineage-based regionalization within the SGZ comparable to that observed in the SVZ. NSCs in the SGZ are primarily derived from the cortical hem, where Wnt-responsive Axin2^+^ NSCs emerge during embryonic development and remain broadly distributed in the adult DG (Bowman et al., 2013). In addition, Shh-responsive Gli1^+^ NSCs originate from the ventral hippocampus during late gestation and gradually migrate during development to replenish the dorsal hippocampal region (Li et al., 2013). Hopx^+^ NSCs are thought to arise from a common embryonic progenitor pool and continue to sustain neurogenesis into adulthood (Berg et al., 2019). However, imaging and lineage-tracing studies have not shown that these NSC subpopulations form stable spatial fate domains within the adult DG (Pilz et al., 2018). This feature may be related to the relatively concentrated embryonic origin of the DG as well as its highly folded structural organization.

Despite the absence of clearly defined fate domains, the DG still exhibits functional state differences along the longitudinal axis of the hippocampus. Previous studies have shown that NSCs in the dorsal DG generally display higher levels of proliferation and activation than those in the ventral region, and that newly generated neurons also tend to mature more rapidly in the dorsal DG (Amaral and Witter, 1989; Jinno, 2011). In addition, regional differences are observed in responses to external stimuli: environmental enrichment and learning-related stimuli preferentially enhance neurogenesis in the dorsal DG, whereas chronic stress more readily suppresses neurogenesis in the ventral DG (Erginousakis and Papatheodoropoulos, 2025). These observations suggest that although the SGZ does not form stable lineage-based spatial domains, functional gradients may still exist within this niche.

**Figure 2:**
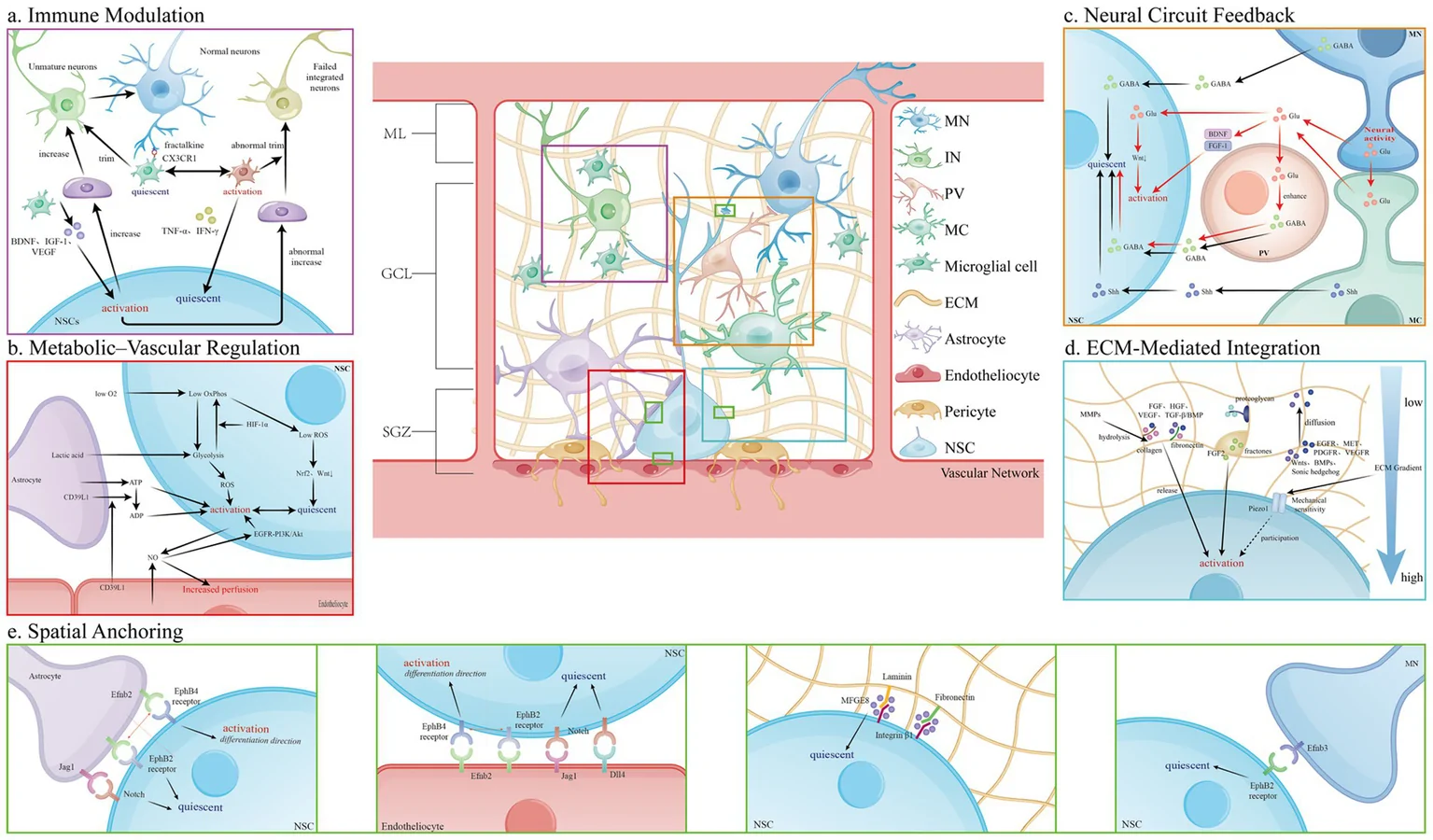
A modular framework for the regulation of neural stem cell states within the SGZ neurogenic niche. Neural stem cells (NSCs) in the subgranular zone (SGZ) are regulated by a multi-layered niche environment composed of multiple interacting cellular and structural components. Rather than being governed by a single dominant signal or a strictly hierarchical architecture, NSC behavior emerges from the integration of several interrelated regulatory dimensions. These regulatory processes can be conceptually organized into five major modules: spatial anchoring, metabolic–vascular regulation, neural circuit feedback, immune regulation, and extracellular matrix (ECM)-mediated signal integration. At the systems level, these modules interact through multidirectional feedback and coordinated dynamics, collectively shaping a constrained regulatory environment that defines the range of accessible NSC states. Transitions between quiescence, activation, and differentiation arise from coordinated changes across multiple regulatory dimensions rather than from isolated perturbations. Representative schematic panels illustrate key mechanisms within each module. The spatial anchoring **(e)** defines the physical positioning of NSCs and their response thresholds to external signals through cell–cell contacts and local signal distribution. The metabolic–vascular regulation **(b)** establishes the physiological feasibility of state transitions by regulating oxygen supply, energy substrates, and redox balance. The neural circuit feedback **(c)** provides activity-dependent inputs, dynamically coupling neurogenesis to behavioral states and functional demands. The immune regulation **(a)**, primarily mediated by microglia and glial cells, modulates NSC activation frequency, lineage specification, and integration efficiency through state-dependent signaling. Meanwhile, ECM-mediated signaling **(d)** integrates and reorganizes these inputs at the spatial level by regulating signal distribution, receptor organization, and mechanical context, thereby converting them into localized regulatory cues that can be sensed by NSCs. ADP, adenosine diphosphate; ATP, adenosine triphosphate; BDNF, brain-derived neurotrophic factor; ECM, extracellular matrix; FGF, fibroblast growth factor; GABA, *γ*-aminobutyric acid; GCL, granule cell layer; HIF-1α, hypoxia-inducible factor 1 alpha; IGF-1, insulin-like growth factor 1; IN, immature neuron; MC, mossy cell; MN, mature neuron; MMPs, matrix metalloproteinases; NSC, neural stem cell; PV, parvalbumin-positive interneuron; ROS, reactive oxygen species; SGZ, subgranular zone; Shh, sonic hedgehog; VEGF, vascular endothelial growth factor.

At the level of local tissue organization, the SGZ neurogenic niche exhibits a highly specialized cellular architecture (Figure 2). NSCs are arranged in a band-like distribution along the base of the granule cell layer (GCL). Their cell bodies closely appose the GCL, while basal processes extend downward toward the vascular-rich hilus (Devasthali et al., 2025). After vascular branches from the hippocampal fissure enter the DG, they pass vertically through the GCL; the capillaries then turn approximately 90° and run parallel to the SGZ, forming a densely interwoven vascular network (Gómez-Gaviro et al., 2012). The processes of NSCs can directly associate with the vascular basement membrane and interact with endothelial cells and pericytes, thereby forming a typical vascular-associated niche structure (Otsuki and Brand, 2017). Astrocytes are widely distributed throughout the SGZ and GCL; their processes both ensheath NSCs and connect to vascular structures via endfeet, spatially establishing an important network that integrates vascular, neuronal, and stem cell signaling (Verkhratsky et al., 2016; Willis et al., 2022). Microglia are scattered within the SGZ and contribute to the regulation of neurogenesis by clearing apoptotic cells, participating in synaptic pruning, and secreting a variety of regulatory factors (Sierra et al., 2010; Gemma and Bachstetter, 2013). In addition, mature dentate granule neurons (Qin et al., 2026), local interneurons (Song et al., 2012), and mossy cells (MCs) (Yeh et al., 2018) also participate in shaping the local regulatory environment. The ECM serves as both a structural scaffold and a signaling interface, playing an important role in maintaining the stability of this complex cellular network (Ioannidis et al., 2021). Together, these cellular components form a highly integrated local microenvironmental network.

Recent studies further suggest that these longitudinal functional differences may be associated with multiple structural features of the SGZ niche. First, at the level of vascular organization, imaging studies indicate the presence of differences in blood supply along the longitudinal axis of the hippocampus. The anterior circulation, via the anterior choroidal artery, provides relatively direct perfusion to the medial temporal lobe, whereas more posterior regions rely primarily on more indirect vascular routes (Vockert et al., 2021). Such differences in perfusion patterns may influence local oxygen tension, the availability of metabolic substrates, and the exchange of vascular-derived factors, thereby creating distinct metabolic environments for neurogenic activity in different regions.

Second, at the level of neural circuitry, the dorsal hippocampus is primarily connected with sensory and higher-order cognitive cortical areas, whereas the ventral hippocampus receives projections from structures associated with emotion and stress, including the amygdala, hypothalamus, and nucleus accumbens (Moser and Moser, 1998; Strange et al., 2014). These differences in input patterns suggest that neurogenesis in different regions may be regulated by distinct types of neural activity signals.

Third, at the level of the molecular microenvironment, transcriptomic studies have revealed stable gene expression gradients along the longitudinal axis of the hippocampus. A range of genes, including those related to the ECM, show continuous variation along the anterior–posterior axis. Importantly, these differences primarily reflect changes in intracellular expression levels rather than alterations in the relative proportions of cell types (Vogel et al., 2020). This finding suggests that local cells may shape distinct microenvironmental features along the longitudinal axis through differential secretion of ECM-related molecules and other regulatory factors.

Taken together, current evidence indicates that the SGZ lacks the stable spatial fate domains observed in the SVZ. Nevertheless, its niche architecture still exhibits a degree of functional regional variation, which may be jointly influenced by multiple factors, including vascular supply, neural circuit inputs, and the molecular microenvironment. Notably, most of these factors vary in a continuous gradient rather than forming discrete spatial boundaries. Accordingly, the SGZ is more likely to represent a continuous neurogenic niche in which regulation is primarily state-dependent. Within this framework, NSCs lack stable spatial fate anchors, and their behavior depends largely on the dynamic integration of local microenvironmental signals. Consequently, the strength, duration, and combinatorial patterns of microenvironmental signals may play critical roles in determining the direction and magnitude of NSC state transitions. Interpreting these key regulatory factors from the perspective of niche architecture will therefore help to further clarify the overall regulatory logic governing SGZ neurogenesis.

## 3 Regulatory mechanisms of the SGZ niche

As one of the major sites of adult neurogenesis, the SGZ contains a tightly integrated niche composed of diverse cellular populations and structural components. Although the molecular and cellular mechanisms underlying each individual component remain incompletely characterized, the functional interconnections and interdependencies among different niche elements have become increasingly apparent. Current evidence generally suggests that the NSC states in the SGZ emerge from the coordinated influence of multiple layers of niche regulation.

The transition from quiescence to activation represents one of the central yet most complex regulatory events governing adult NSC behavior. Although numerous extracellular cues, including inflammatory mediators, metabolic signals, neurotransmitters, and extracellular matrix-derived factors, have been implicated in this process, their relative contributions and interactions remain incompletely understood. Most current studies have focused on individual signaling pathways or specific niche components, providing important mechanistic insights but offering only a partial understanding of how these diverse inputs are integrated within the SGZ microenvironment. For example, Belenguer et al. (2021) demonstrated that systemic inflammation can prime adult NSCs through TNF-*α* receptor signaling, increasing their responsiveness to subsequent activation cues (Ottone et al., 2014). These findings suggest that NSC activation is unlikely to be governed by any single signaling pathway. Instead, it is more likely to emerge from the coordinated integration of multiple niche-derived regulatory inputs, with the relative contribution of each regulatory dimension varying according to physiological and pathological contexts.

The regulatory mechanisms of the SGZ niche can be conceptualized as comprising several interrelated structural–functional dimensions. These include spatial anchoring, metabolic permission, neural circuit feedback, maintenance of immune homeostasis, and interfaces for signal integration (Figure 2). Together, these dimensions form a multilayered niche regulatory framework that shapes and directs NSC state transitions.

### 3.1 Spatial signals governing quiescence and activation: localization and threshold regulation of NSCs

NSCs in the SGZ do not exist in isolation but are embedded within a highly specialized niche composed of vascular endothelial cells, pericytes, astrocytes, and ECM. Through multiple forms of structural contact, NSCs maintain their spatial positioning. At the same time, these interactions provide sustained contact-dependent signals that define activation thresholds required for maintaining long-term quiescence and enabling reversible activation under specific conditions. Here, the activation threshold refers to the signaling requirements necessary for NSCs to transition from a stable quiescent state to an activatable state. Current evidence indicates that stable structural interactions between NSCs and their niche components constitute the fundamental basis for the maintenance of NSC quiescence.

Under homeostatic conditions, vascular endothelial cells represent one of the primary sources of contact-dependent regulation. Endothelial cells continuously express the transmembrane ligands ephrinB2 (Efnb2) and Jagged1 (Jag1), which are associated with the Eph and Notch signaling pathways. Through direct cell–cell contact, these ligands activate inhibitory signaling in NSCs, thereby restricting excessive proliferation and maintaining the stability of the stem cell pool. *In vivo* conditional knockout studies have shown that specific deletion of Efnb2 or Jag1 leads to a marked increase in NSCs proliferative activity in the short term, followed by sustained depletion of the stem cell pool. This finding suggests that such contact-dependent signals play an indispensable role in imposing “threshold constraints” that prevent aberrant activation (Belenguer et al., 2021). In addition, endothelial cells also express the Notch ligand Dll4, which is particularly enriched in arterial vessels and in regions of angiogenesis, such as endothelial tip cells (Noguera-Troise et al., 2006). Dll4 enhances local Notch signaling intensity within the vascular-adjacent microenvironment, thereby further elevating the activation threshold of nearby NSCs and rendering perivascular regions more inhibitory (Ridgway et al., 2006).

Astrocytic endfeet ensheath NSCs and provide structural support within the niche. They also express Jagged1 and ephrin-B2, thereby contributing to the restriction of excessive NSCs proliferation and the maintenance of stem cell pool stability (Wilhelmsson et al., 2012; Ashton et al., 2012). Notably, these contact-dependent signals are not purely inhibitory. Under specific conditions, astrocytes can also participate in fate regulation following NSC activation. For example, in co-culture systems, astrocyte-derived ephrin-B2 promotes neuronal lineage differentiation through interaction with the EphB4 receptor on NSCs. Importantly, EphB4 expression is low in quiescent NSCs but is markedly upregulated upon activation (Ashton et al., 2012). These findings suggest that structural contacts not only regulate the threshold for NSC activation, but may also modulate downstream signal responsiveness through state-dependent changes in receptor expression, thereby influencing lineage commitment.

Beyond blood vessels and astrocytes, neuron-derived contact signals also contribute to the maintenance of NSC quiescence. Mature DG dentate granule cells (DGCs) express ephrin-B3, which can interact with EphB2 receptors on NSCs to suppress aberrant activation. This suggests that the structural state of neural networks can feed back to the stem cell level and participate in the maintenance of their homeostasis (Dong et al., 2019).

The ECM participates in signal transduction through integrin-mediated adhesion, thereby constituting another key mechanism of spatial regulation. Laminin interacts with integrin receptors on the surface of NSCs, contributing to the maintenance of their adhesive state and the regulation of proliferative behavior. Integrin-linked kinase (ILK) further plays an important role in stabilizing cell adhesion and maintaining cellular morphology (Porcheri et al., 2014; Radakovits et al., 2009). In addition, the soluble ECM-associated molecule milk fat globule–epidermal growth factor 8 (MFGE8) is enriched in quiescent NSCs (Zhou et al., 2018). Through β1 integrin–mediated cell–ECM adhesion, MFGE8 is thought to support stable interactions between NSCs and vascular-associated matrix components (Cheyuo et al., 2019), while reinforcing adhesion-dependent signaling to maintain quiescence and suppress aberrant activation (Li et al., 2024). Researchers have also demonstrated that dynamic remodeling of the ECM in the SVZ and protease-mediated degradation and reorganization can affect the local microenvironment in a manner that leads to neurogenesis (Nakajo et al., 2026). The precise mechanisms in the SGZ remain incompletely understood. Nevertheless, these findings suggest that the ECM is not merely a static anchor but may actively adjust NSC activation thresholds through its own dynamic remodeling.

Pericytes also maintain close spatial proximity to NSCs. Although direct evidence for contact-dependent signaling mechanisms by which pericytes regulate NSC behavior remains limited, as key components of the neurovascular unit, they play essential roles in maintaining vascular integrity and microenvironmental homeostasis. In doing so, they provide a structural basis for the stable transmission of vascular-derived signals and indirectly support the maintenance of NSC quiescence (Bell et al., 2010).

Taken together, structural signals that depend on cell–cell contact and spatial proximity are characterized by slow dynamics and high stability, forming a basal regulatory layer within the NSC niche. By continuously providing inhibitory or constraining inputs, these signals establish the activation threshold of NSCs and define the range of their responsiveness to external cues. On this basis, such signals can be conceptualized as a gating mechanism: only when other regulatory modules—such as metabolic state, neural activity, or immune signals—reach sufficient strength to overcome this threshold do NSCs undergo state transitions. Notably, during NSC activation, remodeling of the receptor expression profile may partially relieve these structural constraints and confer selective responsiveness to specific anchoring cues, thereby guiding subsequent lineage decisions within the framework of spatial restriction. Thus, structural contact–mediated signals not only maintain NSC spatial positioning and quiescent stability, but also define the response pattern.

### 3.2 Metabolic responses in neurovascular coupling: dynamic regulation involving NSCs

In the SGZ, the dense vascular network is closely associated with NSCs. Structurally, NSCs in the SGZ are closely apposed to vascular endothelial cells, pericytes, and astrocytes, together constituting a local neurovascular unit that provides the anatomical basis for neurovascular coupling and its associated metabolic regulation (Apple and Kokovay, 2017). This organization not only ensures a continuous supply of blood flow, oxygen, and metabolic substrates to NSCs, but also enables NSCs to sense local metabolic and redox microenvironment changes. In this context, vascular signals may act as upstream drivers of NSC metabolic states and subsequent fate transitions.

On this basis, NSCs in the SGZ appear to respond to local variations in oxygen availability and metabolic state in a gradient-dependent manner. The SGZ as a whole resides in a relatively hypoxic environment (Chatzi et al., 2015). Under quiescent conditions, low levels of reactive oxygen species (ROS) help maintain the self-renewal capacity of NSCs while suppressing premature differentiation (Sunatani et al., 2024). However, when oxygen availability or metabolic conditions change, NSC activation and differentiation behaviors shift accordingly. *In vitro* studies have shown that reducing oxygen concentration to physiologically hypoxic levels can significantly enhance the proliferative capacity of embryonic NSCs (Arvidsson et al., 2002). Under pathological conditions, such as the severe hypoxia induced by transient cerebral ischemia, adult NSCs can be activated and neurogenesis is promoted (Hase et al., 2024). These findings indicate that oxygen levels, by modulating metabolic state and the redox environment, exert differential effects on NSC fate decisions across distinct ranges.

At the molecular level, hypoxia-inducible factors (HIFs) serve as key nodes linking changes in oxygen availability to metabolic reprogramming. HIFs promote the expression of glycolysis-related genes while suppressing oxidative phosphorylation, thereby maintaining low levels of ROS and supporting NSCs in an undifferentiated state (Tomita et al., 2003). As oxygen availability increases, HIF-1α is progressively degraded, and cellular metabolism shifts from glycolysis toward oxidative phosphorylation, accompanied by enhanced mitochondrial biogenesis and increased tricarboxylic acid cycle activity (Daly et al., 2021; Garone et al., 2024). This metabolic transition improves the efficiency of ATP production, providing energetic support for differentiating cells, while also inducing dynamic changes in ROS levels. Notably, ROS in this context are not merely metabolic byproducts but also activate pathways such as Nrf2 and Wnt, promoting cell cycle exit and the initiation of neurogenic programs (Kosaka et al., 2010; Haack et al., 2015). Conversely, excessive oxidative stress disrupts mitochondrial quality control and metabolic reprogramming, leading to impaired mitochondrial dynamics, premature NSC differentiation, and progressive depletion of the stem cell pool. Accordingly, redox homeostasis is not only essential for metabolic adaptation but also for preserving mitochondrial integrity and sustaining long-term NSC function through coordinated redox-responsive signaling pathways, including Nrf2 and JNK (Torrisi et al., 2025). These findings suggest that metabolic state can regulate the sensitivity of NSCs to differentiation cues by reshaping the redox signaling environment.

Neurovascular coupling is further amplified by blood flow–dependent signaling, in which nitric oxide (NO) serves as a key mediator. Produced by both endothelial and neurogenic cells, NO generation is in turn tightly regulated by the local redox state (Gileadi, 2014). On the one hand, NO induces vasodilation by activating soluble guanylate cyclase (sGC) and upregulating the cGMP/PKG signaling pathway, which reduces intracellular Ca^2+^ levels in pericytes and vascular smooth muscle cells. This process increases local perfusion and rapidly enhances the delivery of oxygen and metabolic substrates (Borysova and Burdyga, 2015). On the other hand, NO also functions directly as a redox signaling molecule in the regulation of NSCs, exhibiting negative regulatory effects. Inhibition of NO synthesis has been shown to enhance neurogenesis (Packer et al., 2003; Park et al., 2001), whereas NO donors suppress NSC proliferation (Torroglosa et al., 2007). Mechanistically, these effects are mediated in part through inhibition of the EGFR–PI3K/Akt signaling axis, as well as reversible S-nitrosylation of EGFR (Murillo-Carretero et al., 2009). Together, these findings indicate that NO exerts dual roles in both vascular regulation and intracellular signaling, acting as a critical node that links macroscopic changes in blood flow and metabolic supply with microscopic fate decisions of NSCs.

Beyond oxygen availability and blood flow, NSC function is also regulated at a broader level through energy substrates and purinergic signaling. Astrocytes play a key intermediary role in this context. Through the “lactate shuttle,” they supply metabolic substrates to local cells, thereby supporting NSC proliferation and long-term maintenance (Álvarez et al., 2016; (Li et al., 2025)). At the same time, ATP also acts as a paracrine signal regulating NSC behavior, with its effects partly dependent on mechanotransduction mediated by the mechanosensitive channel Piezo1 (Chi et al., 2022). Within the perivascular niche, astrocytes and pericytes express the ectonucleotidase CD39L1, which hydrolyzes ATP to ADP (Braun et al., 2003), thereby establishing an efficient paracrine purinergic signaling microenvironment in neurogenic regions (Mishra et al., 2006). ADP promotes the self-renewal and expansion of both mouse and human NSCs via P2Y purinergic receptors (Lin et al., 2007). These findings suggest that NSCs, glial cells, and the vascular system cooperatively form a metabolic signaling circuit centered on ATP/ADP dynamics.

In addition, neural activity can indirectly influence NSC behavior by modulating this coupled system. Neuropeptides released by local interneurons regulate astrocytic glutamatergic and purinergic cascades, thereby linking neural activity with metabolic state and blood flow regulation (Chi et al., 2022), and further reinforcing the integrative role of neurovascular coupling.

Notably, NSCs exert feedback regulation on this coupled system. Through NO-mediated paracrine signaling, NSCs induce the vascular expression of pro-angiogenic factors including vascular endothelial growth factor (VEGF) and brain-derived neurotrophic factor (BDNF); concurrently, NSCs themselves also serve as a direct source of these factors. In doing so, NSCs promote angiogenesis and support the metabolic demands of NSC actions (Li et al., 2006; Iyer et al., 2007). These findings indicate that the relationship between NSCs and the vascular system represents a bidirectional interaction, with NSCs functioning as active regulatory nodes within the niche.

Taken together, NSCs and vascular components in the SGZ form a dynamic regulatory system through neurovascular coupling. This coupling is based on the interplay between blood flow-driven substrate supply and NSC metabolic demands and is further influenced by metabolic reprogramming and redox signaling. In this framework, a regulatory mechanism centered on “metabolic demand–supply matching” is established. Although the precise mechanisms remain to be fully elucidated, current evidence suggests that metabolic support itself also functions as a feedback signal influencing NSC fate decisions. Under physiological conditions, this coupling mechanism maintains a dynamic balance between preserving NSC quiescence and meeting the energetic demands associated with activation. In contrast, under pathological conditions, disruption of this system may lead to impairment of the neurogenic microenvironment, thereby contributing to the pathogenesis and progression of various neurological disorders.

### 3.3 Activity-dependent feedback from neural circuits: maintenance and triggering of NSC fate by local networks

NSCs in the SGZ are located at the base of the GCL in the DG. This anatomical positioning places them directly within a highly localized neural circuit composed of mature DGCs, inhibitory interneurons (INs), and MCs (Llorente et al., 2022). This anatomical arrangement exposes NSCs to local neurotransmitter signals and places them under strong activity-dependent regulation.

Under homeostatic conditions, local circuits maintain NSC quiescence primarily through an inhibitory tone. Mature DGCs and INs continuously release *γ*-aminobutyric acid (GABA), establishing a stable inhibitory signaling environment within the SGZ. As NSCs lack conventional synaptic GABAergic inputs, this signaling is mainly mediated through spillover transmission or locally elevated GABA concentrations, which act on NSCs to increase activation thresholds and restrict inappropriate cell cycle entry (Tozuka et al., 2005). At the same time, the structural and functional stability of mature DGCs themselves is critical for maintaining this inhibitory milieu. Studies have shown that aberrant expression of Klf9 leads to reduced excitability and impaired dendritic architecture in mature DGCs, accompanied by abnormal activation of NSCs and enhanced neurogenesis; restoration of normal Klf9 expression correspondingly returns NSC states to homeostasis (McAvoy et al., 2016). These findings indicate that the structural integrity of mature neurons and the stability of local network activity are essential prerequisites for maintaining the balanced state of NSCs in the SGZ.

On this basis, neural activity does not simply “promote” NSCs but instead regulates their activation conditions by reshaping the balance between inhibition and excitation within local circuits, as well as the associated secretory signaling environment. When circuit activity increases, mature DGCs can reduce the expression of the Wnt signaling inhibitor sFRP3, thereby relieving suppression of the Wnt pathway and promoting the activation of quiescent NSCs, as well as subsequent neuronal differentiation and maturation (Jang et al., 2013). In addition, neural activity can exert longer-lasting effects through epigenetic mechanisms. For example, activity-induced upregulation of Gadd45b promotes the demethylation and expression of genes such as BDNF and fibroblast growth factor-1 (FGF-1), thereby extending the pro-neurogenic effects of transient activity over longer timescales (Ma et al., 2009). Together, these findings indicate that neural circuit activation can not only rapidly modulate NSC states but also convert transient activity signals into sustained molecular memory.

MCs and INs together constitute key integrative nodes within this regulatory process. As major excitatory relays in the hilus, MCs play dual roles. On the one hand, they form monosynaptic excitatory connections and directly release glutamate, thereby integrating and amplifying local network excitatory signals to promote the activation of NSCs or nearby progenitor cells (Scharfman and Myers, 2013). On the other hand, MCs can recruit INs, such as parvalbumin-positive (PV^+^) interneurons, to enhance local GABAergic tone, thereby reinforcing the maintenance of NSC quiescence (Yeh et al., 2018). In addition, MC-derived Sonic hedgehog (Shh) signaling contributes to the sustained regulation of NSC quiescent state (Gonzalez-Reyes et al., 2019). Among these, PV^+^ interneurons play a particularly prominent role in maintaining NSC quiescence by suppressing aberrant activation via both GABAergic signaling and the ErbB4–BDNF–TrkB signaling axis, and by restoring neurogenic balance under conditions such as stress (Bao et al., 2017). At the same time, these interneurons integrate long-range inputs from regions such as the medial septum, allowing behavioral states (e.g., arousal and locomotion) to influence SGZ neurogenesis at the circuit level (Bao et al., 2017). Overall, interneuron networks stabilize neural activity output through precise regulation of the excitation–inhibition balance, thereby preventing NSCs from over-responding to transient signals.

Overall, local neural circuits establish a regulatory framework within the SGZ niche in which triggering and constraint operate in parallel. Under physiological homeostasis, an intact network architecture and stable neuronal activity ensure sustained inhibitory input, maintaining NSCs in a quiescent state and thereby preserving the long-term stability of the stem cell pool. In contrast, when behavioral activation or environmental changes induce circuit remodeling, the release of inhibitory constraints together with the introduction of pro-differentiation signals promotes the activation and differentiation of NSCs. At the same time, excitation–inhibition integration within the circuit ensures that these processes remain controlled in both magnitude and duration, thereby enabling orderly neurogenic output. Thus, neural circuits not only determine whether NSCs are activated, but also shape NSCs by regulating the timing, magnitude, and cross-layer coupling of niche signals.

At present, the specific roles of different subtypes of INs and projection-defined interneurons in maintaining NSC homeostasis and mediating activation transitions remain to be systematically elucidated. However, accumulating evidence indicates that circuit-level regulation is coupled with other regulatory layers. For example, local circuit activity can rapidly modulate blood flow and endothelial signaling through neurovascular coupling, thereby amplifying its regulatory influence on the NSC niche (Noguchi et al., 2023; Shen et al., 2019). Therefore, understanding how local neural circuits operate dynamically within the broader niche framework, and how they coordinate with other regulatory layers, represents a central challenge in elucidating the mechanisms underlying adult SGZ neurogenesis.

### 3.4 Immune state–driven process regulation: microglia-mediated guidance of NSC fate

Microglia are resident immune cells within the neurogenic niche, characterized by highly dynamic motility, enabling them to establish direct contacts with multiple cell types and rapidly respond to local microenvironmental changes (Paris et al., 2018; Nimmerjahn et al., 2005; Davalos et al., 2005). In the adult DG, microglia are primarily distributed within the molecular layer and at the GCL–hilus boundary, and exhibit relatively high turnover rates, suggesting that they function as active regulatory components continuously involved in maintaining niche homeostasis (Sierra et al., 2010; Askew et al., 2017). Current evidence indicates that microglia serve as key intermediaries in NSC immune regulation, connecting the local immune environment to neurogenesis through state-dependent signaling.

Microglia, the primary immune sentinels of the CNS, are not phenotypically fixed. Rather, their state is continually calibrated by a broad spectrum of niche inputs, including vascular components, pericytes, neuronal activity, astrocytes, and metabolic cues (Zhao et al., 2025). In turn, microglia actively feed back to each of these elements, forming a bidirectional regulatory interface (Zhao et al., 2025). The functional state of microglia is determined by their transcriptional profile, morphological characteristics, and secretory repertoire, all of which can dynamically shift in response to microenvironmental cues (Godeanu and Cătălin, 2025). This highly context-dependent mode of regulation positions microglia within the SGZ niche as both executors of immune modulation and integrative nodes for multiple signaling inputs.

Single-cell transcriptomic analyses indicate that microglia in the adult DG predominantly exhibit a low-inflammatory, homeostasis-associated transcriptional profile, rather than remaining in a persistently activated inflammatory state (Artegiani et al., 2017). Further studies demonstrate that disruption of microglial function in either direction can impair adult neurogenesis: excessive activation is detrimental to the maintenance and maturation of newborn neurons (Fawcett et al., 2019; Monje et al., 2003), whereas partial genetic ablation of microglia in adult mice likewise results in a reduction in the number of adult-born neuroblasts (Kreisel et al., 2019). Together, these findings suggest that microglia are indispensable components of neurogenic homeostasis, with regulatory effects that are highly dependent on their functional phenotype, incoming signals, and the surrounding microenvironmental context.

Transitions between microglial states influence cytokine release profiles, thereby modulating NSC behavior. Under inflammatory or injury-associated activation state, microglia secrete pro-inflammatory cytokines such as TNF-*α* and IFN-*γ*, which suppress NSC activation and impair the maturation of newly generated neurons. In contrast, under physiological homeostasis or repair-associated functional states, microglia release trophic factors including BDNF, insulin-like growth factor-1 (IGF-1), and VEGF, which support NSC activation and the maintenance of neurogenesis (Kohman et al., 2012; Mäkelä et al., 2010; Bernardino et al., 2008). Importantly, the functional phenotype of microglia itself reflects the overall state of the local immune environment. Dynamic transitions between microglial activation states determine the relative weighting of pro-inflammatory and trophic signals, thereby modulating NSC states and differentiation bias at the systems level.

In addition, microglia contribute to niche regulation through downstream cellular clearance. By efficiently removing aberrant or apoptotic cells while preserving viable cell populations, they recycle essential molecular components and thereby support subsequent NSC activation and differentiation. Multiple studies have shown that, under physiological conditions, NSCs themselves undergo relatively little apoptosis (Zhang et al., 2025; Ryu et al., 2016), whereas programmed cell death is predominantly observed at the level of amplifying progenitors and early neuroblasts. Microglia maintain niche homeostasis through the efficient phagocytosis of apoptotic cells and cellular debris, a process that is accompanied by the release of diverse neurotrophic factors and cytokines, which in turn feedback to regulate NSC activation and differentiation (Diaz-Aparicio et al., 2020; Beccari et al., 2017). Functional studies further indicate that chronic TAM receptor-mediated phagocytic impairment suppresses NSC activation, whereas acute MerTK downregulation-induced phagocytic dysfunction may provoke NSC overactivation (Diaz-Aparicio et al., 2020). Collectively, these findings suggest that the sustained appropriateness of microglial phagocytic activity, rather than its absolute intensity, represents a key parameter for maintaining NSCs within an optimal activation range. Through continuous clearance of apoptotic cells and cellular debris, microglia not only preserve structural homeostasis of the local microenvironment, but also generate a phagocytosis-associated secretome that releases neurotrophic and immunomodulatory factors, thereby exerting feedback regulation on NSC activation and differentiation. In this way, microglia convert the “state of cellular turnover” into signaling outputs that can be sensed by NSCs.

Microglial states can also contribute to structural remodeling of local neural circuits through synaptic pruning. Both developmental and adult studies have shown that microglia are capable of engulfing presynaptic terminals and dendritic spines, with a preferential elimination of low-activity synaptic inputs, thereby contributing to activity-dependent circuit refinement (Paolicelli et al., 2011; Schafer et al., 2012; Weinhard et al., 2018). In the DG, ultrastructural evidence demonstrates that microglial processes ensheathing DGCs participate in structural remodeling within the hilus–GCL region, suggesting that synaptic pruning may indirectly regulate adult neurogenesis by influencing the integration of newborn neurons and local circuit activity (Ribak et al., 2009). Conversely, neural circuit activity also regulates microglial functional states. Neuron-derived fractalkine, acting on CX3CR1 receptors expressed by microglia, contributes to the maintenance of adult hippocampal neurogenesis, whereas CX3CR1 deficiency is associated with reduced proliferation, fewer adult-born DGCs, and altered microglial activation states (Vukovic et al., 2012; Bolós et al., 2018). However, direct evidence that synaptic pruning determines NSC activation fate remains lacking and the effects are more likely mediated indirectly through modulation of the excitation–inhibition balance within the circuit.

Overall, the functional phenotype of microglia reflects the overall state of the local immune environment. Dynamic transitions between distinct activation states determine the relative weighting of pro-inflammatory and trophic signals, thereby regulating NSC activation thresholds and differentiation bias and imposing directional constraints on neurogenesis. On this basis, microglia couple their internal state to specific effector functions. On the one hand, through the sustained clearance of apoptotic cells and cellular debris, together with the release of a phagocytosis-associated secretome, they convert the “state of cellular turnover” into feedback signals that regulate NSC behavior. On the other hand, through synaptic pruning, microglia participate in circuit remodeling and indirectly modulate the excitation–inhibition balance, thereby incorporating neural activity states into the regulation of NSCs.

Thus, microglia constitute a key regulatory interface within the SGZ that integrates immune state, cellular turnover, and circuit structural information. This forms a regulatory module centered on “state sensing–structural clearance–signal feedback,” which continuously shapes NSC behavioral trajectories and ultimately determines the magnitude and persistence of neurogenic output. This regulatory framework further suggests that microglia act as critical intermediaries that assess niche conditions and adjust neurogenic output strategies accordingly.

### 3.5 From signals to interface: ECM as a spatial organizational platform for SGZ regulation

In addition to direct cell–cell interactions, many regulatory signals within the neurogenic niche exist in the form of diffusible factors, whose range and duration of action largely depend on the organization of the ECM. The ECM provides the fundamental structural support and cellular positioning framework for the SGZ niche. It is composed of diverse glycoproteins, proteoglycans, and cell adhesion molecules, and is widely distributed among the cellular components of the neurogenic niche (Barros et al., 2011). Core ECM components include tenascins, reelin, laminins, heparan sulfate proteoglycans, and their corresponding receptors such as integrins. These molecules have been shown to regulate progenitor cell proliferation, migration, and lineage differentiation during both embryonic and adult neurogenesis (Long and Huttner, 2019). The ECM not only serves as a structural scaffold of the SGZ but also functions as a critical interface linking “molecular signals” to “spatial regulation.” By modulating signal distribution, receptor organization, and the mechanical microenvironment, the ECM helps organize diffusible molecular signals into a spatially structured environment that can be sensed by NSCs.

Within the SGZ niche, the ECM helps organize signaling factors from multiple cellular sources by shaping their spatial distribution. Variations and potential conflicts among these signals, arising from individual cellular states, are resolved through binding, local concentration, and controlled release, thereby forming spatially defined signaling microdomains. In this way, diffusible signals are transformed into localized inputs that can be precisely sensed by NSCs. The spatial organizational properties of the ECM are particularly evident in certain neurogenic regions. Studies have shown that in the SVZ, ECM microstructures enriched in heparan sulfate proteoglycans, known as fractones, are capable of capturing fibroblast growth factor-2 (FGF-2) and establishing localized high-concentration signaling microdomains, thereby promoting NSC expansion (Kerever et al., 2007; Mercier, 2016). Although morphologically distinct fractone structures have not been clearly identified in the DG, functionally analogous ECM microdomains are likely to exist and may similarly contribute to the spatial localization and coordinated regulation of paracrine signaling.

At the level of signal presentation, multiple ECM components—including fibronectin, collagen, and proteoglycans—can bind and locally concentrate growth factors such as FGF, HGF, VEGF, and members of the TGF-*β*/BMP family, thereby restricting their diffusion range while prolonging their local bioactivity (Zhu et al., 1999; Wang et al., 2008). Correspondingly, proteolytic systems such as matrix metalloproteinases (MMPs) can remodel the ECM and release sequestered ligands, converting them into soluble, bioactive signals that are accessible to cells (Iyer et al., 2007). In addition, a variety of receptor tyrosine kinases (e.g., EGFR, MET, PDGFR, VEGFR) (Kim et al., 2011) and cytokine-related receptors (including Notch, FGFs, Wnts, BMPs, and Shh pathways) (Brizzi et al., 2012) have been shown to form physical or functional complexes with integrins. Through these interactions, the ECM enables coordinated signal amplification and retention.

At the mechanical level, the ECM further regulates NSC behavior through its physical properties. Atomic force microscopy measurements have revealed a pronounced gradient of tissue stiffness increasing from the SGZ toward the GCL (Luque et al., 2016). *In vitro* studies demonstrate that adult NSCs can sense changes in ECM stiffness and adjust their mechanical properties and proliferative potential accordingly (Rammensee et al., 2017). The mechanosensitive ion channel Piezo1 serves as a key mediator of this process, with its activity modulated by ECM stiffness. Piezo1 is required for mechanically induced currents and proliferative responses in human NSCs (Pathak et al., 2014). Downstream, interactions between Yap1 and β-catenin, as well as Rho GTPase signaling, have been implicated in transducing Piezo1-mediated mechanical signals into transcriptional programs (Rammensee et al., 2017; Keung et al., 2011). In addition, Piezo1 activity plays an important role in regulating the nuclear localization of Yap1 (Pathak et al., 2014). However, whether these mechanotransduction pathways play a decisive role in adult DG NSCs *in vivo* remains to be directly established.

Overall, the ECM plays a critical role in spatial organization during intercellular signal transmission within the SGZ niche. Through its spatial organization and mechanical gradients, the ECM establishes a physical regulatory network in which NSCs are embedded, thereby constraining and selectively shaping signal delivery. The regulatory effects of the ECM on NSCs can be summarized at three levels. First, it modulates the spatial accessibility of signaling inputs by locally concentrating and releasing diffusible factors. Second, it organizes signal transduction through integrins and associated receptor complexes. Third, it provides mechanical cues through its structural and physical properties. Through the coordinated action of these three mechanisms, the ECM spatially filters signals, organizes receptor complexes, and encodes mechanical states, thereby transforming heterogeneous, multi-source molecular inputs into spatially oriented and functionally specific regulatory signals. In this way, it defines the “effective information set” accessible to NSCs. Accordingly, the ECM serves as an intermediary linking complex niche signals to NSC fate decisions.

Because the ECM integrates signals from multiple cellular sources, its regulatory effects on NSCs are highly context-dependent. As a result, the specific effects of individual ECM components on NSC state transitions in vivo are often difficult to delineate in isolation, and their precise spatial organization and dynamic remodeling mechanisms remain to be systematically characterized. In contrast, under pathological conditions, disruptions in ECM structure or composition frequently lead to global imbalance of niche signals, thereby exerting more pronounced effects on NSC functional states.

In summary, NSC fate in the adult DG SGZ is not determined by any single signal or cell type within the niche, but rather emerges from a multi-layered regulatory network. Current evidence suggests that spatial architecture, blood flow and metabolic permissiveness, neural circuit activity, immune homeostasis, and the ECM-mediated integrative interface can be conceptualized as five coordinated regulatory modules that collectively constrain and direct NSC state transitions and neurogenic output at different levels.

Within this framework, the spatial module defines the physical positioning of NSCs and their response thresholds to external signals through cell–cell contacts and local signal distribution. The metabolic–vascular module establishes the physiological feasibility of state transitions by regulating oxygen supply, energy substrates, and redox balance. The neural circuit module provides activity-dependent inputs, dynamically coupling neurogenesis to behavioral states and functional demands. The immune regulatory module, primarily mediated by microglia and glial cells, modulates NSC activation frequency, lineage specification, and integration efficiency through state-dependent signaling. Meanwhile, the ECM module integrates and reorganizes these inputs at the spatial level by regulating signal distribution, receptor organization, and mechanical context, thereby converting them into localized regulatory cues that can be sensed by NSCs.

At the systems level, these modules interact through multidirectional feedback and coordinated dynamics, integrating structural constraints, metabolic states, neural activity, immune signals, and molecular inputs into a stable yet constrained input environment, effectively defining a “state space” within which NSCs operate. External stimuli or intrinsic fluctuations perturb one or more of these modules, triggering cascading adjustments across the coupled system. In most cases, transitions in NSC states appear to result from coordinated changes across multiple regulatory dimensions, rather than from isolated perturbations. This multi-factorial regulatory architecture ensures the robustness of adult neurogenesis under physiological conditions, yet renders it vulnerable to system-wide state shifts when confronted with chronic pathological imbalances.

However, it remains unclear how these regulatory modules are hierarchically organized, how feedback operates between them, and how their activities are coordinated over time. The internal logic within each module is also not fully resolved. Moreover, much of the current understanding of SGZ niche regulation is derived from animal models and *in vitro* studies, whereas the complexity and limited accessibility of the human neurogenic niche have substantially constrained direct investigation. Although recent studies using human post-mortem tissue, single-cell transcriptomics, and spatial profiling have provided valuable insights into the cellular composition and disease-associated alterations of the human SGZ, direct validation of the coordinated interactions among these regulatory modules remains limited. Nevertheless, accumulating evidence from human neurodegenerative diseases, together with findings from experimental models, has begun to reveal conserved alterations in the SGZ niche, providing an important foundation for understanding its regulatory mechanisms. Further studies in the human hippocampus will be essential to refine our understanding of SGZ niche dynamics and clarify how these regulatory relationships operate under physiological and pathological conditions.

## 4 Parameter resetting of the SGZ regulatory system under pathological conditions

**Figure 3:**
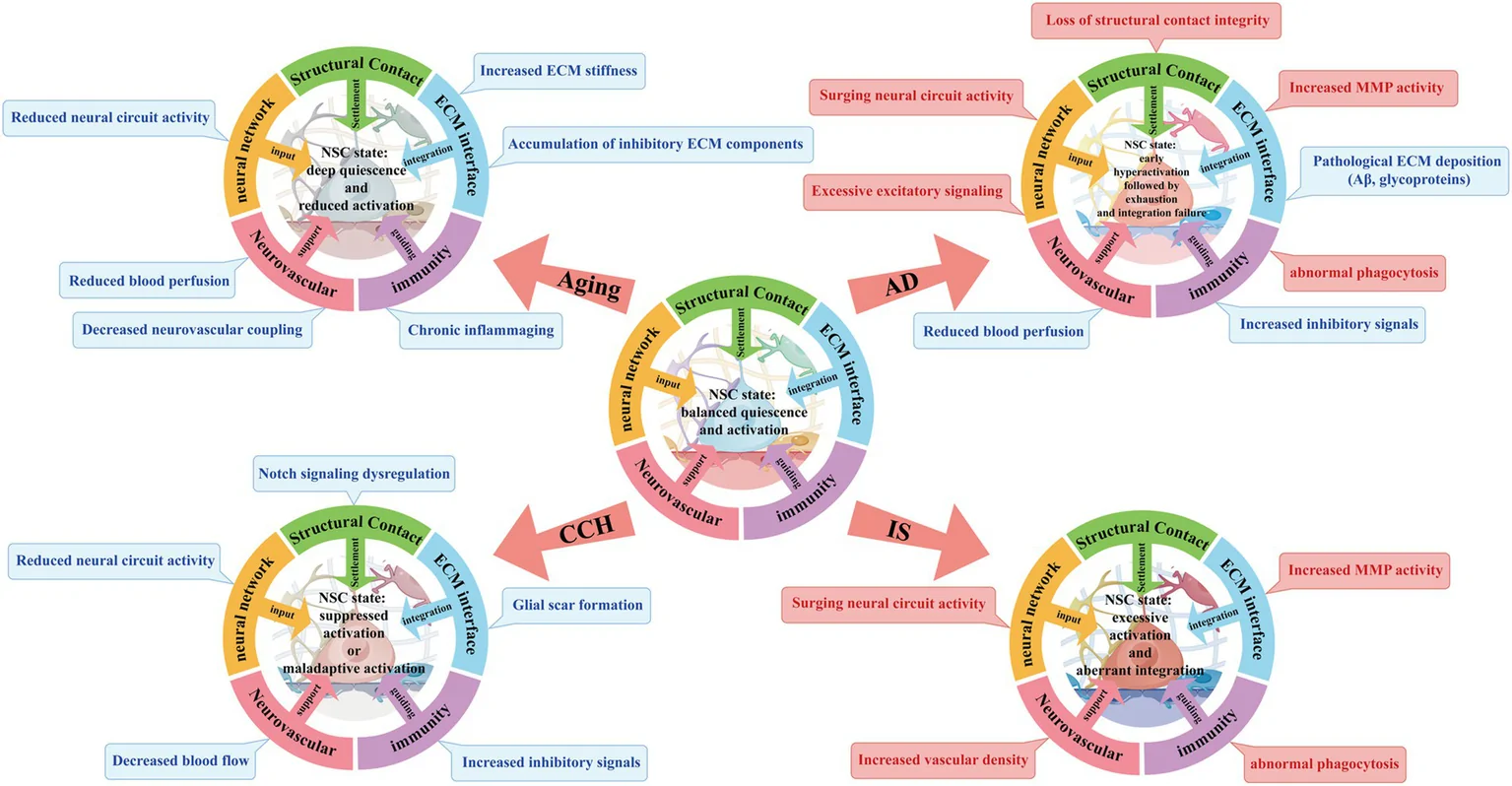
Context-dependent remodeling of the SGZ neurogenic niche across pathological conditions. Pathological conditions induce distinct patterns of subgranular zone (SGZ) niche state shifts, reflecting context-dependent reorganization of a distributed regulatory system. Rather than representing uniform suppression or activation, these states arise from specific combinations of module-level perturbations that reshape neural stem cell (NSC) behavior in different directions. Importantly, these alterations often involve partially opposing or competing regulatory inputs, such that distinct niche components may exert conflicting influences on NSC activation, quiescence, or differentiation. In the figure, the blue color indicates the pathological changes that inhibit or suppress neural development, while the red color represents the pathological changes that activate or disrupt neural development. In aging, reduced neural circuit activity, impaired neurovascular coupling, chronic inflammation, and increased extracellular matrix (ECM) stiffness collectively promote deep quiescence and reduced NSC activation. In Alzheimer’s disease, increased neural circuit hyperactivity, pathological ECM deposition, aberrant immune activation, and disruption of structural contact contribute to early NSC hyperactivation followed by exhaustion and integration failure. In chronic cerebral hypoperfusion (CCH), reduced blood flow, impaired vascular support, glial scar formation, and increased inhibitory signaling suppress NSC activation or lead to maladaptive activation. In ischemic stroke, excessive neural circuit activity, vascular remodeling, immune activation, and ECM degradation drive excessive NSC activation and aberrant integration. Together, these patterns illustrate that NSC state transitions under pathological conditions emerge from coordinated yet not always concordant alterations across multiple regulatory dimensions, reflecting system reorganization of the neurogenic niche.

In aging, neurodegenerative diseases, and acute brain injury, the coordinated interactions among regulatory layers are disrupted in distinct patterns and to varying extents, driving the SGZ niche from a stable, adaptable state toward functional imbalance (Figure 3; Table 1). For this reason, re-examining the regulatory mechanisms of SGZ neurogenesis from a pathological perspective not only facilitates understanding of disease-associated alterations, but also provides a theoretical entry point for dissecting the causal contributions and functional boundaries of each regulatory module within the overall framework.

**Table 1:** Remodeling of the SGZ neurogenic niche in representative neurological conditions and its impact on NSC status.

Disease	Stage/Progression	Key Niche Module alterations					NSC response
		Spatial anchoring	Metabolic–vascular	Neural circuit	Immune regulation	ECM remodeling	
Aging	Chronic aging process characterized by progressive niche aging, reduced metabolic support, circuit hypoactivity, chronic inflammation, and ECM remodeling.	Vascular remodeling; perivascular dysfunction; astrocytic scaffold decline; altered NSC contact stability unclear (Katsimpardi et al., 2014)	Capillary density↓; cerebral blood flow↓; neurovascular coupling↓; metabolic substrate supply↓ (Silva-Vargas et al., 2016)	Synaptic plasticity↓; mature/newborn neurons↓; interneuron activity↓; GABA/glutamate-related feedback↓ (Cao et al., 2024)	Inflammaging; SASP↑; BBB integrity↓; microglial priming; IL-6/TNF-α/IL-1β↑; excessive phagocytosis (Bennett et al., 2024; Trinchero et al., 2019; Chinta et al., 2015; van Deursen, 2014; Muñoz-Espín and Serrano, 2014; Cutler et al., 2025)	Hippocampal stiffness↑; CSPG accumulation; perineuronal net remodeling; TIMP2↓; Reelin↓ (Solano Fonseca et al., 2016; Ryu et al., 2021; Ferreira et al., 2023)	Deep quiescence; reduced activation potential
Alzheimer’s disease (AD)	Aβ/tau accumulation → synaptic dysfunction/network hyperactivity → chronic neuroinflammation/gliosis → vascular–metabolic impairment → ECM remodeling, forming a self-amplifying niche destabilization loop.	Jagged1↓/Notch signaling↓; weakened NSC–astrocyte/endothelial contact; ADAM protease–Notch disruption; Wnt/Notch/integrin signal presentation impaired (Fu et al., 2019; De Strooper and Karran, 2016; Marathe et al., 2017; Kirschen et al., 2018; Li et al., 2013)	BBB permeability↑; capillary degeneration; cerebral perfusion↓; energy/nutrient supply↓; peripheral inflammatory infiltration↑ (Gonzalez-Reyes et al., 2019; Zhuang et al., 2015)	Early hippocampal hyperexcitability/epileptiform activity; Aβ-induced synaptic hyperactivity; PV^+^ interneuron dysfunction; GABAergic regulation↓; later circuit degeneration (Moreno-Jiménez et al., 2019; Cerasuolo et al., 2023)	Aβ/tau-induced microglial and astrocytic activation; IL-1β/TNF-α/IL-18/IFN-γ↑; CCL11↑; chronic inflammatory inhibition of proliferation/differentiation (Hijazi et al., 2023; Sung et al., 2020)	Reelin↓; ApoE4/Aβ/tau-related ECM signaling disruption; MMP activity changes; glycoprotein deposition; perineuronal net remodeling; PV^+^ network dysfunction (Kim et al., 2021; Yi et al., 2024; Radosinska and Radosinska, 2025; de Vries et al., 2025)	Early hyperactivation followed by exhaustion
Chronic cerebral hypoperfusion (CCH)	Sustained hypoperfusion/small vessel pathology → neurovascular stress → metabolic insufficiency → circuit dysfunction → chronic inflammation → ECM remodeling	Vascular pathology; Notch3 dysfunction; possible Notch/contact-dependent signaling disruption; altered vascular ligand and integrin-mediated signaling, direct evidence limited (Shibata et al., 2004)	BBB dysfunction; neurovascular coupling impairment; energy/trophic factor supply↓; metabolic disturbance; oxidative stress↑ (Bouisset et al., 2025; Fraga et al., 2023)	CA1 vulnerability; impaired signal transmission; memory deficits; reduced SGZ synaptic input and activity-dependent stimulation (Tian et al., 2022; Melani et al., 2010; Farkas et al., 2006)	Microglial/astrocytic activation; IL-1β/TNF-α↑; oxidative/toxic signals↑; inflammatory suppression of proliferation and neuronal differentiation; glial lineage bias (Fraga et al., 2023; Ohtaki et al., 2006; Qian et al., 2024)	Collagen/fibronectin/inhibitory proteoglycan deposition; glial scar–like remodeling; disrupted diffusible signal presentation and adhesion-mediated cues (Shibata et al., 2004)	Suppressed or maladaptive activation
Ischemic stroke (IS)	Acute ischemia/excitotoxicity → systemic vascular–trophic signaling and chronic hypoxia → network hyperexcitability → glial inflammation → ECM remodeling → maladaptive neurogenic amplification	Vascular remodeling; vascular density↑; altered NSC–endothelial contact; Notch-related contact signaling likely affected, direct SGZ evidence limited (Cuartero et al., 2021; Zhang et al., 2014)	Mild hypoperfusion/chronic hypoxia; vascular trophic support↑; VEGF/FGF-2/IGF-1/BDNF↑; sustained metabolic–angiogenic remodeling (Niv et al., 2012; Cuartero et al., 2021; Zhang et al., 2014; Lin et al., 2019; Yoshimura et al., 2001; Yoshimura et al., 2003; Yan et al., 2006)	Excitotoxicity; cortical spreading depolarization; extracellular K^+^/glutamate↑; NMDA receptor overactivation; GABAergic inhibition↓; hippocampal hyperexcitability (Chen et al., 2005; Kramer et al., 2016; Mehta et al., 2013; Wang et al., 2011)	Long-term microglial/astrocytic activation; inflammatory cytokines and ROS↑; glial-state changes disrupting newborn neuron integration (Schmitt et al., 2024; Lippmann, 2024)	SDF-1α/MCP-1/MMPs↑; ECM remodeling; altered neuroblast migration, positioning, and newborn neuron integration (Wang et al., 2024)	Excessive activation followed by aberrant integration

The listed alterations summarize representative changes in the SGZ neurogenic niche reported in the studies discussed in the text. Arrows indicate increased (↑) or decreased (↓) expression, activity, abundance, or functional level.

Aβ, amyloid-β; AD, Alzheimer’s disease; ADAM, a disintegrin and metalloproteinase; ApoE4, apolipoprotein E ε4; BBB, blood–brain barrier; BDNF, brain-derived neurotrophic factor; CCH, chronic cerebral hypoperfusion; CCL11, C-C motif chemokine ligand 11; CSPG, chondroitin sulfate proteoglycan; DG, dentate gyrus; ECM, extracellular matrix; FGF-2, fibroblast growth factor 2; GABA, γ-aminobutyric acid; IGF-1, insulin-like growth factor 1; IFN-γ, interferon-γ; IL, interleukin; IS, ischemic stroke; MCP-1, monocyte chemoattractant protein-1; MMPs, matrix metalloproteinases; NMDA, N-methyl-D-aspartate; NSC, neural stem cell; PV, parvalbumin; ROS, reactive oxygen species; SASP, senescence-associated secretory phenotype; SDF-1α, stromal cell-derived factor-1α; SGZ, subgranular zone; TIMP2, tissue inhibitor of metalloproteinases 2; TNF-α, tumor necrosis factor-α; VEGF, vascular endothelial growth factor.

### 4.1 Aging: chronic parameter dysregulation

A large body of evidence consistently shows that adult DG neurogenesis declines significantly during aging. However, this reduction is not primarily driven by irreversible depletion of NSCs, but is more prominently characterized by a decreased probability of activation and an increased proportion of cells in a quiescent state. Transcriptomic and single-cell sequencing analyses of NSC molecular profiles have revealed that, in aged individuals, the proportion of NSCs expressing quiescence-associated signatures is markedly increased, with many entering a state of “deep quiescence” (Kalamakis et al., 2019; Apostolopoulou et al., 2017; Ziebell et al., 2018). Further demonstrated that aged NSCs exhibit greater resistance to activation signals, but once this barrier is overcome, their proliferative and differentiation capacities are largely comparable to those of young NSCs (Pathak et al., 2014). This observation is consistent with findings that external stimuli, such as physical exercise, can partially restore neurogenesis in aged individuals (Lugert et al., 2010). Collectively, aging does not abolish the intrinsic neurogenic potential of NSCs, but rather reconfigures the regulatory landscape by elevating activation barriers and deepening quiescent states, effectively restricting the range of neurogenesis.

This decline in activation probability largely arises from the coordinated dysregulation of multiple niche components during aging. Single-cell transcriptomic analyses indicate that, although differences in lineage-determining gene expression between young and aged NSCs are relatively limited, other cell types within the niche exhibit markedly different sensitivities to aging. Studies based on “transcriptomic aging clocks” have shown that microglia, oligodendrocyte precursor cells, and endothelial cells display more pronounced molecular aging signatures, whereas NSCs exhibit comparatively delayed changes (Buckley et al., 2023). These findings suggest that aging preferentially impacts the external regulatory environment of NSCs rather than the stem cells themselves.

Consistent with this view, both heterochronic parabiosis experiments (Katsimpardi et al., 2014) and interventions involving age-switched choroid plexus secretomes (Silva-Vargas et al., 2016) demonstrate that aged NSCs can regain activation and neurogenic capacity when exposed to a youthful niche environment. Together, these results indicate that the constraints imposed by aging on NSCs primarily reflect alterations in niche parameters, rather than an intrinsic loss of stem cell potential.

At present, relatively few studies have directly addressed how aging affects the spatial contact architecture of the NSC niche. During aging, structural alterations such as vascular remodeling, dysfunction of perivascular cells, neuronal morphological changes, and a decline in the scaffold function of astrocytes are well documented (Cao et al., 2024). However, it remains unclear how these changes affect the stability of NSC contacts with blood vessels and neighboring cells, and how this in turn alters local signal presentation and spatial permissiveness.

In addition, the aged hippocampus exhibits reduced capillary density, decreased cerebral blood flow, and impaired neurovascular coupling efficiency (Bennett et al., 2024). These changes constrain the energy supply and metabolic substrate availability required for NSC activation, and may further influence cell cycle entry by altering local oxygen tension and the metabolic microenvironment.

Neural circuit activity serves as a major source of external input regulating SGZ neurogenesis. During aging, hippocampal networks exhibit reduced synaptic plasticity, decreased numbers of both newly generated and mature neurons, and overall network dysfunction (Trinchero et al., 2019). Concurrently, the activity of local circuits involving interneurons is significantly diminished, leading to reduced levels of activity-dependent regulatory signals—such as GABA and glutamate—received by NSCs. The attenuation of activity-dependent feedback may further impair the responsiveness of NSCs to external stimuli.

Aging brain tissue also exhibits the characteristic features of “inflammaging.” Senescent cells display increased secretory activity, known as the senescence-associated secretory phenotype (SASP), releasing a wide range of pro-inflammatory cytokines, chemokines, and proteases into the extracellular environment (Chinta et al., 2015). In parallel, decline in the blood–brain barrier (BBB) integrity may permit infiltration of peripheral inflammatory factors into the central nervous system, collectively contributing to a chronic inflammatory microenvironment (van Deursen, 2014; Muñoz-Espín and Serrano, 2014). Microglia undergo a shift from a homeostatic surveillance state toward a mildly pro-inflammatory phenotype. This aberrant activation not only exacerbates inflammation but may also disrupt NSC pool stability and reduce neurogenesis (Cutler et al., 2025). Multiple inflammatory mediators, including IL-6, TNF-*α*, and IL-1*β*, exert inhibitory effects on NSC function (Solano Fonseca et al., 2016). Notably, emerging evidence suggests that excessive microglial phagocytic activity in aging brain may be partially independent of inflammatory signaling, such that suppression of inflammation alone is insufficient to fully prevent microglia-mediated clearance of NSCs (Ryu et al., 2021).

Aging is also accompanied by continuous remodeling of the ECM network within the SGZ. Studies have shown that the overall stiffness of the aged hippocampus increases, alongside complex alterations in ECM organization. In particular, inhibitory molecules such as chondroitin sulfate proteoglycans (CSPGs) accumulate abnormally within specific structures, including perineuronal nets (Ferreira et al., 2023). These changes may influence cell adhesion properties as well as the local presentation of signaling molecules, thereby altering how NSCs perceive and respond to external cues. In addition, levels of the ECM-associated regulator TIMP2 decline with aging (Durakoglugil, 2025), while reduced expression of Reelin has been linked to decreased NSC proliferation and differentiation capacity (Knopman et al., 2021). Collectively, these findings suggest that ECM remodeling during aging may regulate NSC states by reshaping both the signaling interface and the mechanical properties of the niche.

Overall, aging is accompanied by a gradual and systemic shift in multiple niche parameters within the SGZ. Under the combined influence of reduced blood flow and metabolic support, diminished circuit-derived activity feedback, enhanced immune-inflammatory signaling, and ECM remodeling, NSCs are more likely to remain in a quiescent state that deviates from the normal range of physiological plasticity. Although direct evidence remains limited, weakened structural support may also contribute to this low-output state. At the systems level, the coordinated alteration of these regulatory dimensions elevates the activation threshold of NSCs and constrains their effective response window, thereby stabilizing adult neurogenesis in the SGZ at a persistently low-output state. Importantly, this state remains partially reversible, suggesting that aging primarily alters regulatory constraints rather than irreversibly impairing intrinsic NSC potential.

### 4.2 AD: pathological amplification of niche regulation imbalance

Alzheimer’s disease (AD) is the most common form of dementia and is characterized by progressive cognitive decline along with emotional and behavioral disturbances (Wander and Song, 2021). Its core pathological features include the accumulation of amyloid-β (Aβ) plaques and abnormal hyperphosphorylation of the microtubule-associated protein tau. These pathological processes subsequently result in synaptic dysfunction, chronic neuroinflammation, and neuronal loss (Coupé et al., 2019). In the human brain, the hippocampus is among the earliest and most severely affected regions in AD (Ming and Song, 2011). Adult neurogenesis in the SGZ of DG, which serves as a key source of newly generated neurons, is closely associated with learning, memory, and emotional regulation (Tincer et al., 2016).

Accordingly, preserving and restoring neurogenesis in the SGZ has been proposed as a potential strategy for alleviating cognitive deficits in AD. Current evidence indicates that NSCs in the SGZ undergo cumulative alterations as the AD progresses, including aberrant differentiation, excessive activation, and eventual functional exhaustion. In both human AD patients and multiple animal models, abnormalities in NSC activation patterns have been observed, characterized by an increased proportion of quiescent cells, shifts in lineage bias, and a greater tendency to differentiate into non-neuronal cell types (Siddiqui et al., 2023; Moreno-Jiménez et al., 2019; Fu et al., 2019). During the early stages of AD, even before overt Aβ plaque formation, a transient increase in neural progenitor cell numbers has been reported in the SGZ. This phenomenon is often interpreted as a compensatory attempt at repair. However, such aberrant activation is frequently accompanied by accelerated depletion of the NSC pool and contribute to the progression of subsequent neurodegenerative processes (Liu et al., 2023).

This concept is further supported by transgenic models. For example, in the 3xTg mouse model, deficits in neurogenesis emerge at very early postnatal stages, well before the onset of classical pathological deposition and behavioral abnormalities. Studies have shown that this model exhibits a reduction in neural stem/progenitor cell numbers, impaired proliferative capacity, and decreased generation of new neurons, accompanied by hippocampal atrophy. Notably, significant remodeling of gene expression profiles occurs as early as within the first postnatal month, particularly involving signaling pathways such as Notch and Wnt that are closely associated with NSC fate determination (De Strooper and Karran, 2016).

Although the precise reasons for the high sensitivity of the SGZ to AD pathology remain incompletely understood, accumulating evidence suggests that, regardless of genetic background, the combined accumulation of Aβ and tau can profoundly reshape the neurogenic niche through multiple mechanisms. The pathophysiological processes of AD induce cell-autonomous alterations, chronic neuroinflammation, gliosis, and neural circuit dysfunction (Marathe et al., 2017), thereby driving systemic dysregulation across multiple key regulatory modules of the neurogenic niche, including contact-dependent signaling, vascular and metabolic support, neural circuit activity, immune homeostasis, and the extracellular matrix interface.

At the level of contact-dependent signaling, AD markedly disrupts the direct regulatory interactions between NSCs and neighboring cells. Under physiological conditions, Notch signaling—mediated through cell–cell contact—plays a central role in maintaining long-term stability of the NSC pool. In AD, however, expression of its ligand Jagged1 is reduced, leading to impaired Notch pathway activity and a diminished capacity of NSCs to sustain homeostatic self-renewal (Kirschen et al., 2018). Concurrently, gliosis and vascular structural damage reduce the effective contact area between NSCs and surrounding astrocytes and endothelial cells, thereby weakening the spatial presentation and local integration of key signals such as Wnt, Notch, and integrin-mediated pathways (Li et al., 2013). In addition, altered activity of ADAM family proteases in AD can affect the cleavage and activation of Notch receptors, further disrupting contact-dependent signal transduction at the molecular level (Zhuang et al., 2015; Cerasuolo et al., 2023). Collectively, these changes compromise the contact-mediated regulatory framework upon which NSCs depend, and may contribute to both the aberrant activation observed in early AD and the subsequent failure to maintain the NSC pool over time.

At the vascular and metabolic level, cerebrovascular dysfunction occurs at early stages of AD, including increased permeability of the BBB, capillary degeneration, and reduced cerebral blood perfusion (Hijazi et al., 2023). These changes not only limit the supply of energy and nutrients to the SGZ, but also facilitate the infiltration of peripheral inflammatory factors and immune cells into brain tissue, thereby constraining NSC activation (Noguchi et al., 2023).

At the level of neural circuits, early AD is often accompanied by hippocampal network hyperexcitability and epileptiform activity. Aβ-induced synaptic hyperexcitability and metabolic stress can directly damage the NSC pool. Studies have shown that, in APP transgenic models, abnormal neuronal discharges significantly accelerate the depletion of SGZ NSCs and exacerbate memory deficits (Liu et al., 2023). Meanwhile, dysfunction of INs—particularly PV^+^ neurons—reduces GABAergic regulation that is essential for maintaining NSC quiescence, leading to an imbalance in activity-dependent signaling within the NSC microenvironment (Sung et al., 2020). As the disease progresses, neural circuits gradually shift from early hyperexcitotoxicity to widespread degeneration, and these two stages continuously suppress effective NSC activation through distinct mechanisms.

At the immune level, Aβ oligomers and tau aggregates rapidly activate microglia and astrocytes, inducing sustained release of pro-inflammatory cytokines (such as IL-1β, TNF-*α*, IL-18, IFN-*γ*) and chemokines (such as CCL11) (Kim et al., 2021; Yi et al., 2024). These molecules directly inhibit NSC proliferation and neuronal differentiation, and reduce the survival rate of newborn neurons, making it difficult for aberrantly activated neurogenesis to translate into functional compensation (Yi et al., 2024).

The ECM in the DG also undergoes significant remodeling in AD. Reelin, an extracellular protein closely associated with neuronal migration, synaptic plasticity, and neurogenesis, is markedly downregulated in AD, and its signaling abnormalities interact with ApoE4, Aβ, and tau pathologies, thereby impairing the migration, integration, and functional maturation of newborn neurons (Radosinska and Radosinska, 2025). Additionally, MMPs activity and glycoprotein deposition alter the physical and molecular properties of the SGZ microenvironment, further compromising effective interactions between NSCs and their niche (de Vries et al., 2025). Furthermore, perineuronal nets, specialized extracellular matrix structures surrounding neurons, exhibit more pronounced alterations in AD (Bouisset et al., 2025), which in turn affect signaling within interneuron networks and contribute to PV^+^ neuron dysfunction, representing one of the factors underlying aberrant neural network activity (Bouisset et al., 2025).

Overall, the progressive accumulation of systemic pathological alterations during the course of AD leads to profound dysregulation of the neurogenic niche in the SGZ of the hippocampal DG, driving NSCs into a persistently disturbed pathological regulatory state. At early stages of the disease, changes in spatial structure and contact-dependent signaling weaken the long-term maintenance capacity of NSCs, while abnormally enhanced neural circuit activity induces a compensatory yet niche-unsustained overactivation of NSCs. However, this transient increase in neurogenesis cannot be stably maintained under the chronic neuroinflammatory conditions characteristic of AD, and is instead prone to disruption and eventual functional failure. As the disease progresses, the mismatch between cerebral blood perfusion and metabolic demand imposes energy constraints, while persistently elevated neuroinflammatory inhibition, progressive disruption of neural network function, and remodeling of the ECM collectively amplify the detrimental effects on NSC activation and differentiation. Ultimately, these changes drive adult neurogenesis in the SGZ into a low-level steady state and lead to progressive depletion of the NSC pool.

### 4.3 Chronic cerebral hypoperfusion: cascade dysregulation driven by blood flow and supply limitation

CCH and small vessel pathology are core pathological features of vascular dementia (VaD), yet their impact on the neurogenic niche in the SGZ of the hippocampal DG remains insufficiently characterized. Animal studies indicate that sustained hypoperfusion in bilateral carotid artery occlusion models reduces SGZ neurogenesis and is associated with abnormal neuronal morphology and spatial memory deficits (Tian et al., 2022). This impairment of adult hippocampal neurogenesis is primarily attributed to the increased cell death and reduced proliferation of neuroblasts within the SGZ.

From a mechanistic perspective, the chronic hypoperfusion state caused by long-time insufficient cerebral blood perfusion in VaD represents a direct initiating factor for the overall dysregulation of the NSC niche. Under conditions of sustained blood flow restriction, neurovascular coupling in the SGZ is subjected to prolonged stress and gradually becomes impaired, manifested by BBB dysfunction and local metabolic disturbances (such as insufficient energy supply and reduced availability of trophic factors), thereby disrupting the vascular-supported microenvironment upon which NSCs depend (Melani et al., 2010).

At the level of neural circuits, chronic hypoperfusion can also indirectly affect activity-dependent regulation in the SGZ through remote effects. In the 2VO model, neurons in the CA1 region are highly sensitive to hypoperfusion and metabolic stress, exhibiting early impairments in signal transmission accompanied by deficits in memory function (Farkas et al., 2006; Ohtaki et al., 2006; Qian et al., 2024). This reduction in circuit function may decrease synaptic input to the SGZ, thereby reducing the neurotransmitter signals and activity-dependent stimuli received by NSCs, ultimately impairing their activation, proliferation, and the integration of newly generated neurons.

Meanwhile, chronic ischemia can induce sustained abnormal activation of microglia and astrocytes, leading to increased release of pro-inflammatory cytokines (such as IL-1β and TNF-α) and oxidative stress molecules. This inflammatory response not only exacerbates neuronal apoptosis and BBB disruption, but also directly disturbs the immune homeostasis in which NSCs reside (Melani et al., 2010). Cytokines and toxic signals within the pro-inflammatory environment can suppress the proliferative capacity of NSCs and promote their shift toward a glial lineage (Shibata et al., 2004). Notably, studies have shown that microglia are highly activated in the BCAS model (Ehret et al., 2021), which is considered to be one of the important factors affecting neurogenesis.

At the level of contact-dependent signaling, although direct evidence remains limited, vascular pathology provides important implications for the disruption of NSCs. Relevant studies have shown that, in CADASIL (a hereditary small vessel disease caused by Notch3 mutations) models, dysfunction of Notch3 is closely associated with a marked decline in adult neurogenesis (Ekonomou et al., 2012). This observation suggests that, under conditions of vascular pathology, disruption of contact-dependent signaling pathways such as Notch may impair the self-renewal capacity of NSCs and their ability to determine neuronal lineage fate. In addition, alterations in the composition of ligands on the surface of vascular walls and smooth muscle cells may also affect the adhesion and integrin-mediated signaling received by NSCs; however, direct evidence in the context of VaD remains scarce.

Chronic ischemia, together with associated inflammatory responses, can promote aberrant deposition of extracellular matrix components—such as collagens, fibronectin, and inhibitory proteoglycans—often resulting in the formation of glial scar–like structures (Ekonomou et al., 2012). Such ECM remodeling alters both the physical and molecular properties of the SGZ microenvironment, potentially disrupting the local presentation of diffusible signaling factors as well as the integration of adhesion-mediated cues. Consequently, the functional coupling between NSCs and their niche may be further weakened.

Notably, findings from human studies are not entirely consistent with those observed in experimental models. An autopsy study revealed that small vessel disease is associated with a significant, 33–92% increase (*p* < 0.05) in neural progenitor cell numbers within the SVZ and peri-lesional regions, a phenomenon that persisted even in the presence of severe cerebrovascular pathology (Kernie and Parent, 2010). Although a subset of these progenitor cells was capable of differentiating into immature neurons in peri-lesional areas, this response failed to compensate for overall neuronal loss and showed no direct association with cholinergic system dysfunction. These discrepancies may reflect fundamental differences in the magnitude and temporal profile of blood flow impairment. In commonly used animal models, such as carotid artery ligation, cerebral blood flow reduction is typically rapid and pronounced, whereas human small vessel disease often involves more gradual and heterogeneous hypoperfusion. This suggests that NSC responses are highly dependent on the severity, rate of onset, and duration of perfusion deficits.

Overall, CCH and small vessel disease impose a multi-level, cascade-like disruption on the SGZ neurogenic niche through a state characterized by insufficient perfusion coupled with heightened metabolic stress. Reduced blood flow and limited metabolic support increase the burden on NSC survival and proliferation, while impaired neural circuit function diminishes activity-dependent inputs. Concurrently, persistent inflammation establishes a hostile immune microenvironment, and alterations in adhesion-mediated signaling together with ECM remodeling further weaken effective interactions between NSCs and their niche. Under these conditions, NSCs may still exhibit sustained compensatory activation, potentially linked to their participation in neurovascular coupling (see Section 3.2). However, this response is frequently decoupled from effective neuronal differentiation due to concurrent inflammatory suppression and resource constraints. As a result, NSC activation becomes increasingly uncoupled from productive neurogenesis, and the thresholds and regulatory boundaries governing this transition remain to be fully defined.

### 4.4 Ischemic stroke: maladaptive remodeling following severe disruption

Ischemic stroke induces profound and multi-level remodeling of the neurogenic niche in the SGZ of DG. A large body of evidence consistently demonstrates that hippocampal neurogenesis is markedly enhanced following ischemic insult, across both global and focal ischemia models (Arvidsson et al., 2001b; Arvidsson et al., 2001a; Yagita et al., 2001). In models of global ischemia, proliferative activity within the SGZ can increase by up to tenfold (Kee et al., 2001; Türeyen et al., 2004). Similarly, in focal ischemia paradigms such as middle cerebral artery occlusion (MCAO), robust upregulation of neural progenitor proliferation and differentiation is observed in the SGZ, even when the primary lesion is located in cortical regions remote from the hippocampus (Jin et al., 2001; Cuartero et al., 2019). Notably, this increase in neurogenesis is not limited to the ipsilateral hemisphere, but is also observed in the contralateral hippocampus (Walter et al., 2010; Kadam et al., 2009), suggesting that ischemia triggers systemic or long-range signaling mechanisms capable of globally reprogramming the neurogenic niche.

From a temporal perspective, the proliferative response in the SGZ typically emerges approximately one week after stroke, reaches a peak around days 10–14 in various MCAO models, and gradually declines toward baseline levels by 4–5 weeks post-insult (Sharp et al., 2002; Lin et al., 2018). Although a substantial proportion of newly generated cells undergo apoptosis during this period, the surviving cells largely follow the canonical neurogenic trajectory, differentiating into granule neurons. Their somata migrate from the SGZ into the GCL, accompanied by a sequential transition from doublecortin-positive immature stages to calbindin and NeuN-positive mature neuronal phenotypes (Türeyen et al., 2004; Tanaka et al., 2004; Gallucci et al., 2024). This demonstrates that post-stroke neurogenesis does not appear to deviate substantially from the physiological maturation sequence at the phenotypic level.

However, somewhat paradoxically, patients with ischemic stroke often exhibit progressive cognitive decline during long-term follow-up. Post-stroke cognitive impairment and post-stroke dementia are highly prevalent clinically, affecting multiple domains including memory, executive function, attention, and orientation. More than 40% of patients develop significant cognitive deficits after stroke (Ceanga et al., 2019). Importantly, this cognitive deterioration does not strictly correlate with lesion size or direct involvement of hippocampal structures (Ceanga et al., 2019). A substantial body of evidence from both human and animal studies indicates that even when the primary ischemic lesion is located remotely from the temporal lobe, hippocampal function can still be markedly impaired (Schaapsmeerders et al., 2015; Schmitt et al., 2024; Niv et al., 2012). This suggests that chronic dysregulation of distal neurogenic niches, such as the SGZ, plays a critical role in the development of post-stroke cognitive impairment.

This phenomenon may be directly attributable to the excessive generation of aberrant neurons. In one study, retroviral labeling was performed at day 4 following ischemia, and morphological analyses were conducted 6 weeks post-infection. The results showed that approximately 5–10% of newborn neurons exhibited abnormal structural features, including ectopic localization, aberrant orientation of basal dendrites toward the hilus, as well as increased dendritic complexity and total dendritic length (Cuartero et al., 2021). Notably, the incidence of these aberrant phenotypes was closely associated with the initial infarct size, with a significantly higher proportion of abnormal neurons observed in the filament-induced MCAO model compared to the cortical photothrombotic model. Consistently, another study reported that pro-neurogenic interventions, such as voluntary exercise, failed to improve—and instead exacerbated—cognitive impairment during the post-stroke phase. In contrast, selective attenuation of post-stroke neurogenesis in the SGZ markedly improved contextual and spatial memory performance in MCAO mice at the chronic stage (Walter et al., 2010). This finding indicates that the aberrantly enhanced hippocampal neurogenesis following stroke does not constitute a protective reparative response, but rather contributes to the development of long-term cognitive deficits.

In contrast, selective attenuation of post-stroke neurogenesis in the SGZ markedly improved contextual and spatial memory performance in MCAO mice at the chronic stage, indicating that the aberrantly enhanced hippocampal neurogenesis following stroke does not constitute a protective reparative response, but rather contributes to the development of long-term cognitive deficits (Walter et al., 2010).

At present, the mechanisms by which a focal infarct induces functional disturbances in remote regions, such as the hippocampus, remain incompletely understood. However, from the perspective of key regulatory modules within the neurogenic niche, accumulating evidence suggests that such effects may arise through the coordinated involvement of multiple processes, including vascular and metabolic support, neuronal circuit activity, immune responses, and extracellular matrix remodeling.

Persistent low-grade chronic hypoxia following stroke has been proposed as an important background factor driving aberrant hippocampal neurogenesis (Zhang et al., 2014). Ischemic injury progressively exposes NSCs in the SGZ to a microenvironment characterized predominantly by mild hypoperfusion, thereby systemically reshaping the interactions between NSCs and their niche. Under these conditions, this alters the basal state of metabolic supply, vascular signaling, and activity-dependent inputs. Meanwhile, NSC-centered adaptive feedback mechanisms remain continuously engaged (see Section 4.3), sustaining neurogenesis at an adaptive activation state and further amplifying subsequent pathological consequences.

First, ischemic injury induces profound alterations in circulating factors and vascular signaling, thereby reshaping the vascular-associated niche in which NSCs reside. These changes further influence local metabolic supply as well as contact-dependent signaling between NSCs and vascular endothelial cells. Studies have shown that, following ischemia, the number of GFAP^+^ NSCs in the SVZ is significantly increased, accompanied by a marked rise in vascular density over several months, indicating sustained vascular and cellular remodeling of the neurogenic niche (Lin et al., 2019). These alterations are closely associated with the upregulation of multiple trophic and angiogenic factors, including VEGF (Yoshimura et al., 2001), FGF-2 (Yoshimura et al., 2003; Yan et al., 2006), IGF-1 (Chen et al., 2005), and BDNF (Kramer et al., 2016). Although direct evidence in the SGZ remains limited, given the widespread release and systemic origin of these factors following ischemic injury, similar changes are likely to occur within the SGZ, thereby providing metabolic and trophic support for sustained, high-level NSC activation. Meanwhile, as vascular architecture undergoes remodeling, the contact interface between NSCs and endothelial cells is also likely altered. This may in turn affect contact-dependent signaling pathways such as Notch, thereby modulating NSC self-renewal and activation thresholds (Yoshimura et al., 2001).

Second, at the level of neural circuit regulation, abnormal network activity may represent a central driving force underlying excessive hippocampal neurogenesis. During the acute phase of ischemia, excitotoxicity and cortical spreading depolarization lead to elevated extracellular levels of potassium ions and glutamate (Mehta et al., 2013). This environment, on the one hand, promotes aberrant proliferation of immature neural progenitors, while on the other hand exerts toxic effects on mature neurons (Wang et al., 2011). Given that granule cell development is highly dependent on NMDA receptor-mediated activity, excessive activation of NMDA receptors following stroke may result in abnormally increased excitatory synaptic input to newborn neurons, thereby accelerating their excitatory maturation and amplifying circuit imbalance (Lippmann, 2024). Meanwhile, mechanisms that normally suppress mature granule cell activity via GABAergic interneurons become impaired under ischemic conditions, instead driving the hippocampal network into a state of heightened hyperexcitability (Mehta et al., 2013; Wang et al., 2024). Together, these factors lead to an explosive amplification of activity-dependent regulation of NSCs, ultimately resulting in excessive NSC activation.

However, in parallel with these pro-proliferative signals, persistent inflammation and glial reactivity are also present. Ischemia induces long-term activation of microglia and astrocytes, whose release of inflammatory cytokines, ROS, and phenotypic changes in glial states significantly disrupt the precise integration of newborn neurons (Wesley et al., 2017). Morphological tracing studies have shown that, among newly generated granule cells after stroke, the proportion of cells exhibiting ectopic localization, aberrant basal dendrite orientation, and excessive dendritic complexity is markedly increased, with the degree of abnormality positively correlated with the severity of the initial lesion (Cuartero et al., 2021). After integrating into the GCL, these aberrant newborn neurons can further disrupt the organization of the pre-existing neural network.

In addition, at the level of ECM, ischemia-associated inflammatory responses induce profound ECM remodeling, accompanied by the upregulation of multiple chemokines and matrix-regulatory molecules (such as SDF-1α, MCP-1, and MMPs) (Ruan et al., 2015). These changes not only affect the migration and positioning of neuroblasts, but also alter the physical and molecular environment required for the proper integration of newborn neurons.

Taken together, under conditions of extensive tissue damage, ischemic stroke triggers a wave of neurogenesis that is predominantly compensatory in quantity, yet markedly mismatched with post-injury tissue architecture, neural circuit states, and niche conditions. In the acute phase, this response manifests as compensatory activation; however, in the chronic phase, aberrant integration of newborn neurons and persistent circuit imbalance lead to sustained disruption of hippocampal network function, contributing to the progressive cognitive decline observed after stroke. Thus, ischemia-induced neurogenesis should not be viewed as a simple regenerative response, but rather as a form of mismatch-driven remodeling arising from multi-module niche dysregulation. Its defining feature lies in the pronounced dissociation between increased neurogenesis output and failed functional integration.

In summary, different types of central nervous system diseases do not act on NSCs in the SGZ through a single pathway. Rather, they reshape NSC states by altering the coordinated interactions among multiple key regulatory modules, including contact-dependent signaling, metabolic supply, neural activity, immune state, and extracellular matrix organization, ultimately leading to disease-specific patterns of neurogenesis and functional outcomes. This perspective suggests that, whether in the context of endogenous or exogenous NSC activation and therapeutic intervention, attempts to interpret or manipulate NSC behavior in isolation from the neurogenic niche are unlikely to yield stable or predictable outcomes.

Although a comprehensive and systematic understanding of NSC regulatory mechanisms under different pathological conditions remains limited, accumulating evidence from multiple representative disease models has begun to reveal distinct patterns of coordinated multi-module shifts within the neurogenic niche.

During aging, the neurogenic niche undergoes a gradual and sustained functional decline, characterized by reduced blood flow and metabolic support, impaired neurovascular coupling, diminished neural circuit activity, ECM remodeling, and an increased burden of chronic low-grade inflammation. These changes collectively elevate the activation threshold of NSCs and bias them toward a state of deep quiescence, indicating that NSC behavior is highly dependent on the overall supply capacity of the niche. At the same time, this process suggests that neurogenesis, as an energy-demanding and tightly regulated biological process requiring strict quality control, is governed by relatively conservative threshold mechanisms. Such regulation serves to prevent ineffective or aberrant activation under unfavorable conditions.

In AD, pathological factors such as Aβ not only directly impair the neurogenic niche, but also exacerbate its systemic burden by inducing chronic inflammation, synaptic dysfunction, and ECM remodeling, thereby exerting multi-level suppression on NSC activation and differentiation. This process illustrates how a single pathological driver can amplify its biological impact through cross-module interactions, ultimately reshaping the global state of the niche and leading to system-level imbalance. Although NSCs may exhibit a degree of compensatory activation under these conditions, such responses are rarely translated into effective neuroregeneration. Instead, they suggest that, under chronic pathological stress, the adaptive capacity of the niche may become persistently misdirected.

CCH models highlight the fundamental role of blood flow and metabolic supply in regulating NSC behavior. Sustained reduction in perfusion directly constrains the extent and efficiency of neurogenesis, while also revealing the strong coupling among different regulatory modules: metabolic limitation not only affects NSC activity, but also indirectly reshapes its regulatory context by altering neural circuit states and the immune environment. Meanwhile, the observed increase in neural progenitor cell numbers in patients with small vessel disease suggests the presence of compensatory regulation between NSCs and their niche. However, the efficiency of this response, as well as its long-term stability, remains to be further validated.

In contrast, acute ischemic stroke represents a far more disruptive context of niche perturbation. Vascular damage, inflammatory responses, and rapid ECM remodeling act in concert within a short time frame, leading to cascade-like imbalance and maladaptive reconfiguration of the neurogenic niche. This process further underscores that the structural integrity of the niche as a whole, together with its capacity for dynamic adaptation, is critical for maintaining stable neurogenesis and brain function.

Interpreting NSC behavior from the perspective of system-level regulation within the neurogenic niche may provide a coherent framework for understanding the shared mechanisms underlying altered neurogenesis across different neurological diseases. The heterogeneous changes in neurogenesis observed under distinct disease conditions may reflect specific patterns of imbalance within a multi-module regulatory system governing the niche. In this context, disease-specific differences in neurogenesis can be conceptualized as the entry of this multi-module system into distinct “state regimes,” along with their corresponding dynamic trajectories over time. The consistent and pronounced alterations in NSC states across these pathological models further indicate that the neurogenic niche retains a degree of plasticity that is amenable to remodeling and intervention. A clearer understanding of the temporal relationships and causal interactions among these modules may enable more precise control of NSC behavior in the future.

## 5 Conclusion

Adult neurogenesis in the SGZ of the DG is tightly regulated by the neurogenic niche. The spatial organization of NSCs, together with the unique structural architecture of the SGZ, underscores the central role of niche-level regulation in shaping NSC behavioral states. Variations in niche structure underlie the functional diversity of SGZ neurogenesis.

In this review, we propose a conceptual framework in which the regulation of NSCs in the SGZ neurogenic niche can be understood from the perspective of distinct “regulatory modules.” This analytical approach facilitates the disentanglement of key regulatory processes from secondary influences within complex signaling networks, thereby enabling a clearer interpretation of the core structure underlying NSC behavior. Current evidence indicates that multiple key regulatory modules collectively govern the maintenance and state transitions of NSCs within the SGZ niche, including contact-dependent signaling, vascular and metabolic conditions, neural circuit activity, immune homeostasis and microglial states, as well as ECM-mediated signal integration. Importantly, these modules do not operate in isolation; rather, they form a highly interconnected regulatory network that constrains and coordinates NSC state transitions within a dynamically balanced microenvironment.

Within this framework, NSC activation, proliferation, and differentiation can be viewed as the outcome of coordinated interactions among multiple niche regulatory modules. External stimuli or pathological perturbations rarely act through a single signaling pathway; rather, they reshape NSC behavior by altering the relationships and balance among these modules, thereby reconfiguring the niche environment that governs NSC state transitions. Evidence from diverse disease models is broadly consistent with this modular perspective, suggesting that different neurological disorders may correspond to distinct patterns of niche dysregulation. Although pathological remodeling often follows disease-specific temporal trajectories, the sequence and extent of module involvement vary according to the underlying pathology and even different stages of disease progression. Perturbations in one regulatory dimension may subsequently propagate to other modules through reciprocal interactions, resulting in progressive niche remodeling. Consequently, distinct pathological phenotypes are likely to arise from differences in both the pattern and temporal evolution of SGZ microenvironmental alterations. Such dysregulation can ultimately give rise to varying degrees and forms of altered adult hippocampal neurogenesis, and further contribute to diverse structural and functional outcomes in the brain.

Current therapeutic strategies aimed at enhancing adult hippocampal neurogenesis, including endogenous NSC activation and stem cell transplantation, increasingly suggest that successful regeneration cannot rely solely on manipulating NSCs themselves but must also consider the surrounding niche environment. Accumulating evidence indicates that combined approaches targeting inflammation, vascular function, metabolic support, extracellular matrix remodeling, and neural activity generally achieve better outcomes than single-target interventions (Chen et al., 2025; Bao and Song, 2018). However, the SGZ niche represents a highly dynamic and integrated regulatory system in which pathological alterations occur across multiple interacting dimensions. Consequently, identifying the critical niche alterations that drive NSC dysfunction and determining whether coordinated modulation of the niche can precisely redirect NSC behavior toward functional regeneration remain major challenges. We therefore propose that future therapeutic strategies should move beyond single-pathway interventions and instead focus on restoring the integrated regulatory properties of the neurogenic niche.

It should be emphasized that while the modular framework proposed here provides a structured perspective for understanding SGZ niche regulation, it should not be interpreted as reflecting a strictly hierarchical biological organization. In practice, NSC behavior is shaped by the integration of multiple, often competing, niche-derived signals, and current evidence remains insufficient to define a clear causal hierarchy among these regulatory processes. Rather than representing a fixed regulatory architecture, this framework is better understood as an analytical construct that captures key dimensions of niche regulation. Such a perspective may help to organize complex observations, while acknowledging that NSC regulation fundamentally emerges from a distributed and context-dependent system. Future studies integrating human post-mortem analyses, single-cell and spatial omics, and functional experimental models will be essential. Such integrated approaches will uncover novel regulatory mechanisms, refine our understanding of SGZ niche dynamics, and elucidate the orchestration of NSC behavior by the neurogenic microenvironment across physiological and pathological states.

Interpreting NSC behavior from the perspective of integrated niche regulation provides a potential theoretical framework for explaining the diversity of neurogenesis under both physiological and pathological conditions. Further identification of regulatory modules that are preferentially altered in specific disease contexts may offer new avenues for precision interventions targeting the niche. Collectively, we further emphasize that the neurogenic niche, rather than NSCs in isolation, represents the primary actionable unit for therapeutic modulation of adult neurogenesis.

## References

[ref1] Altman J. (1962). Are new neurons formed in the brains of adult mammals? Science 135, 1127–1128. doi: 10.1126/science.135.3509.1127, 13860748

[ref2] Altman J. Das G. D. (1965). Autoradiographic and histological evidence of postnatal hippocampal neurogenesis in rats. J. Comp. Neurol. 124, 319–335. doi: 10.1002/cne.901240303, 5861717

[ref3] Álvarez Z. Hyroššová P. Perales J. C. Alcántara S. (2016). Neuronal progenitor maintenance requires lactate metabolism and PEPCK-M-directed cataplerosis. Cereb. Cortex 26, 1046–1058. doi: 10.1093/cercor/bhu281, 25452568

[ref4] Amaral D. G. Witter M. P. (1989). The three-dimensional organization of the hippocampal formation: a review of anatomical data. Neuroscience 31, 571–591. doi: 10.1016/0306-4522(89)90424-7, 2687721

[ref5] Apostolopoulou M. Kiehl T. R. Winter M. Cardenas De La Hoz E. Boles N. C. Bjornsson C. S. . (2017). Non-monotonic changes in progenitor cell behavior and gene expression during aging of the adult V-SVZ neural stem cell niche. Stem Cell Reports 9, 1931–1947. doi: 10.1016/j.stemcr.2017.10.005, 29129683 PMC5785674

[ref6] Apple D. M. Kokovay E. (2017). Vascular niche contribution to age-associated neural stem cell dysfunction. Am. J. Phys. Heart Circ. Phys. 313, H896–H902. doi: 10.1152/ajpheart.00154.2017, 28801522 PMC5792207

[ref7] Aran S. Golmohammadi M. G. Sagha M. Ghaedi K. (2025). Aging restricts the initial neural patterning potential of developing neural stem and progenitor cells in the adult brain. Front. Aging Neurosci. 16:1498308. doi: 10.3389/fnagi.2024.1498308, 39916688 PMC11798963

[ref8] Artegiani B. Lyubimova A. Muraro M. J. van Es J. H. van Oudenaarden A. Clevers H. (2017). A single-cell RNA sequencing study reveals cellular and molecular dynamics of the hippocampal neurogenic niche. Cell Rep. 21, 3271–3284. doi: 10.1016/j.celrep.2017.11.050, 29241552

[ref9] Arvidsson A. Collin T. Kirik D. Kokaia Z. Lindvall O. (2002). Neuronal replacement from endogenous precursors in the adult brain after stroke. Nat. Med. 8, 963–970. doi: 10.1038/nm747, 12161747

[ref10] Arvidsson A. Kokaia Z. Airaksinen M. S. Saarma M. Lindvall O. (2001a). Stroke induces widespread changes of gene expression for glial cell line-derived neurotrophic factor family receptors in the adult rat brain. Neuroscience 106, 27–41. doi: 10.1016/S0306-4522(01)00268-8, 11564414

[ref11] Arvidsson A. Kokaia Z. Lindvall O. (2001b). N-methyl-D-aspartate receptor-mediated increase of neurogenesis in adult rat dentate gyrus following stroke. Eur. J. Neurosci. 14, 10–18. doi: 10.1046/j.0953-816x.2001.01611.x, 11488944

[ref12] Ashton R. S. Conway A. Pangarkar C. Bergen J. Lim K.-I. Shah P. . (2012). Astrocytes regulate adult hippocampal neurogenesis through ephrin-B signaling. Nat. Neurosci. 15, 1399–1406. doi: 10.1038/nn.3212, 22983209 PMC3458152

[ref13] Askew K. Li K. Olmos-Alonso A. Garcia-Moreno F. Liang Y. Richardson P. . (2017). Coupled proliferation and apoptosis maintain the rapid turnover of microglia in the adult brain. Cell Rep. 18, 391–405. doi: 10.1016/j.celrep.2016.12.041, 28076784 PMC5263237

[ref14] Bao H. Asrican B. Li W. Gu B. Wen Z. Lim S.-A. . (2017). Long-range GABAergic inputs regulate neural stem cell quiescence and control adult hippocampal neurogenesis. Cell Stem Cell 21, 604–617.e5. doi: 10.1016/j.stem.2017.10.003, 29100013 PMC5689456

[ref15] Bao H. Song J. (2018). Treating brain disorders by targeting adult neural stem cells. Trends Mol. Med. 24, 991–1006. doi: 10.1016/j.molmed.2018.10.001, 30447904 PMC6351137

[ref16] Barros C. S. Franco S. J. Müller U. (2011). Extracellular matrix: functions in the nervous system. Cold Spring Harb. Perspect. Biol. 3:a005108. doi: 10.1101/cshperspect.a005108, 21123393 PMC3003458

[ref17] Beccari S. Valero J. Maletic-Savatic M. Sierra A. (2017). A simulation model of neuroprogenitor proliferation dynamics predicts age-related loss of hippocampal neurogenesis but not astrogenesis. Sci. Rep. 7:16528. doi: 10.1038/s41598-017-16466-3, 29184142 PMC5705784

[ref18] Belenguer G. Duart-Abadia P. Jordán-Pla A. Domingo-Muelas A. Blasco-Chamarro L. Ferrón S. R. . (2021). Adult neural stem cells are alerted by systemic inflammation through TNF-α receptor signaling. Cell Stem Cell 28, 285–299.e9. doi: 10.1016/j.stem.2020.10.016, 33207218

[ref19] Bell R. D. Winkler E. A. Sagare A. P. Singh I. LaRue B. Deane R. . (2010). Pericytes control key neurovascular functions and neuronal phenotype in the adult brain and during brain aging. Neuron 68, 409–427. doi: 10.1016/j.neuron.2010.09.043, 21040844 PMC3056408

[ref20] Bennett H. C. Zhang Q. Wu Y.-T. Manjila S. B. Chon U. Shin D. . (2024). Aging drives cerebrovascular network remodeling and functional changes in the mouse brain. Nat. Commun. 15:6398. doi: 10.1038/s41467-024-50559-8, 39080289 PMC11289283

[ref21] Berg D. A. Su Y. Jimenez-Cyrus D. Patel A. Huang N. Morizet D. . (2019). A common embryonic origin of stem cells drives developmental and adult neurogenesis. Cell 177, 654–668.e15. doi: 10.1016/j.cell.2019.02.010, 30929900 PMC6496946

[ref22] Bernardino L. Agasse F. Silva B. Ferreira R. Grade S. Malva J. O. (2008). Tumor necrosis factor-alpha modulates survival, proliferation, and neuronal differentiation in neonatal subventricular zone cell cultures. Stem Cells 26, 2361–2371. doi: 10.1634/stemcells.2007-0914, 18583543

[ref23] Boldrini M. Fulmore C. A. Tartt A. N. Simeon L. R. Pavlova I. Poposka V. . (2018). Human hippocampal neurogenesis persists throughout aging. Cell Stem Cell 22, 589–599.e5. doi: 10.1016/j.stem.2018.03.015, 29625071 PMC5957089

[ref24] Bolós M. Perea J. R. Terreros-Roncal J. Pallas-Bazarra N. Jurado-Arjona J. Ávila J. . (2018). Absence of microglial CX3CR1 impairs the synaptic integration of adult-born hippocampal granule neurons. Brain Behav. Immun. 68, 76–89. doi: 10.1016/j.bbi.2017.10.002, 29017970

[ref25] Borysova L. Burdyga T. (2015). Evidence that NO/cGMP/PKG signalling cascade mediates endothelium-dependent inhibition of IP3R-mediated Ca2+ oscillations in myocytes and pericytes of ureteric microvascular network in situ. Cell Calcium 58, 535–540. doi: 10.1016/j.ceca.2015.08.006, 26344105 PMC4655834

[ref26] Bouisset G. Tixier F. J. Dupak T. Laurino A. Cecconi F. Canto C. . (2025). Enriched environment requires the remodeling of hippocampal perineuronal nets to trigger memory improvement in Alzheimer’s mouse model. iScience 28:113344. doi: 10.1016/j.isci.2025.113344 40917885 PMC12410357

[ref27] Bowman A. N. van Amerongen R. Palmer T. D. Nusse R. (2013). Lineage tracing with Axin2 reveals distinct developmental and adult populations of Wnt/β-catenin-responsive neural stem cells. Proc. Natl. Acad. Sci. USA 110, 7324–7329. doi: 10.1073/pnas.1305411110, 23589866 PMC3645553

[ref28] Braun N. Sévigny J. Mishra S. K. Robson S. C. Barth S. W. Gerstberger R. . (2003). Expression of the ecto-ATPase NTPDase2 in the germinal zones of the developing and adult rat brain. Eur. J. Neurosci. 17, 1355–1364. doi: 10.1046/j.1460-9568.2003.02567.x, 12713638

[ref29] Brizzi M. F. Tarone G. Defilippi P. (2012). Extracellular matrix, integrins, and growth factors as tailors of the stem cell niche. Curr. Opin. Cell Biol. 24, 645–651. doi: 10.1016/j.ceb.2012.07.013, 22898530

[ref30] Buckley M. T. Sun E. D. George B. M. Liu L. Schaum N. Xu L. . (2023). Cell-type-specific aging clocks to quantify aging and rejuvenation in neurogenic regions of the brain. Nature Aging 3, 121–137. doi: 10.1038/s43587-022-00335-4, 37118510 PMC10154228

[ref31] Cao Y. Xu W. Liu Q. (2024). Alterations of the blood-brain barrier during aging. J. Cereb. Blood Flow Metab. 44, 881–895. doi: 10.1177/0271678X241240843, 38513138 PMC11318406

[ref32] Ceanga M. Keiner S. Grünewald B. Haselmann H. Frahm C. Couillard-Després S. . (2019). Stroke accelerates and uncouples intrinsic and synaptic excitability maturation of mouse hippocampal DCX+ adult-born granule cells. J. Neurosci. 39, 3303–3317. doi: 10.1523/JNEUROSCI.3303-17.2018, 30617211 PMC6391573

[ref33] Cerasuolo M. Papa M. Colangelo A. M. Rizzo M. R. (2023). Alzheimer’s disease from the amyloidogenic theory to the puzzling crossroads between vascular, metabolic and energetic maladaptive plasticity. Biomedicine 11:861. doi: 10.3390/biomedicines11030861, 36979840 PMC10045635

[ref34] Chaker Z. Makarouni E. Doetsch F. (2024). The organism as the niche: physiological states crack the code of adult neural stem cell heterogeneity. Annu. Rev. Cell Dev. Biol. 40, 381–406. doi: 10.1146/annurev-cellbio-120320-040213, 38985883

[ref35] Chatzi C. Schnell E. Westbrook G. L. (2015). Localized hypoxia within the subgranular zone determines the early survival of newborn hippocampal granule cells. eLife 4:e08722. doi: 10.7554/eLife.08722 26476335 PMC4714973

[ref36] Chen L. Li Z. Wang W. Zhou Y. Li W. Wang Y. (2025). Adult hippocampal neurogenesis: new avenues for treatment of brain disorders. Stem Cell Reports 20:102600. doi: 10.1016/j.stemcr.2025.102600, 40816272 PMC12447344

[ref37] Chen J. Zacharek A. Zhang C. Jiang H. Li Y. Roberts C. . (2005). Endothelial nitric oxide synthase regulates brain-derived neurotrophic factor expression and neurogenesis after stroke in mice. J. Neurosci. 25, 2366–2375. doi: 10.1523/JNEUROSCI.5071-04.2005, 15745963 PMC2791344

[ref38] Cheyuo C. Aziz M. Wang P. (2019). Neurogenesis in neurodegenerative diseases: role of MFG-E8. Front. Neurosci. 13:569. doi: 10.3389/fnins.2019.00569, 31213977 PMC6558065

[ref39] Chi S. Cui Y. Wang H. Jiang J. Zhang T. Sun S. . (2022). Astrocytic Piezo1-mediated mechanotransduction determines adult neurogenesis and cognitive functions. Neuron 110, 2984–2999.e8. doi: 10.1016/j.neuron.2022.07.010, 35963237

[ref40] Chinta S. J. Woods G. Rane A. Demaria M. Campisi J. Andersen J. K. (2015). Cellular senescence and the aging brain. Exp. Gerontol. 68, 3–7. doi: 10.1016/j.exger.2014.09.018, 25281806 PMC4382436

[ref41] Coupé P. Manjón J. V. Lanuza E. Catheline G. (2019). Lifespan changes of the human brain in Alzheimer’s disease. Sci. Rep. 9:3998. doi: 10.1038/s41598-019-39809-8, 30850617 PMC6408544

[ref42] Cuartero M. I. de la Parra J. Pérez-Ruiz A. Bravo-Ferrer I. Durán-Laforet V. García-Culebras A. . (2019). Abolition of aberrant neurogenesis ameliorates cognitive impairment after stroke in mice. J. Clin. Invest. 129, 1536–1550. doi: 10.1172/JCI120412, 30676325 PMC6436875

[ref43] Cuartero M. I. García-Culebras A. Torres-López C. Medina V. Fraga E. Vázquez-Reyes S. . (2021). Post-stroke neurogenesis: friend or foe? Front. Cell Dev. Biol. 9:657846. doi: 10.3389/fcell.2021.657846, 33834025 PMC8021779

[ref44] Cutler R. Harrison S. Sandoval-Kuhn N. Tomlinson S. Kokovay E. (2025). Age associated increase in microglia inflammation and phagocytosis in the adult neural stem cell niche. bioRxiv. doi: 10.1101/2025.10.10.681517

[ref45] Daly L. A. Brownridge P. J. Batie M. Rocha S. Sée V. Eyers C. E. (2021). Oxygen-dependent changes in binding partners and post-translational modifications regulate the abundance and activity of HIF-1α/2α. Sci. Signal. 14:eabf6685. doi: 10.1126/scisignal.abf6685, 34285132

[ref46] Davalos D. Grutzendler J. Yang G. Kim J. V. Zuo Y. Jung S. . (2005). ATP mediates rapid microglial response to local brain injury in vivo. Nat. Neurosci. 8, 752–758. doi: 10.1038/nn1472, 15895084

[ref47] De Strooper B. Karran E. (2016). The cellular phase of Alzheimer’s disease. Cell 164, 603–615. doi: 10.1016/j.cell.2015.12.056, 26871627

[ref48] de Vries L. E. Bahnerth A. Swaab D. F. Verhaagen J. Carulli D. (2025). Resilience to Alzheimer’s disease associates with alterations in perineuronal nets. Alzheimers Dement. 21:e14504. doi: 10.1002/alz.14504, 39737731 PMC11848190

[ref49] Delgado R. N. Lim D. A. (2015). Embryonic Nkx2.1-expressing neural precursor cells contribute to the regional heterogeneity of adult V-SVZ neural stem cells. Dev. Biol. 407, 265–274. doi: 10.1016/j.ydbio.2015.09.008, 26387477 PMC4663161

[ref50] Devasthali N. Carter I. Saulsbery A. I. Kirby E. D. (2025). Postnatal development of the dentate gyrus vascular niche. Sci. Rep. 15:38550. doi: 10.1038/s41598-025-22591-1, 41188405 PMC12586690

[ref51] Diaz-Aparicio I. Paris I. Sierra-Torre V. Plaza-Zabala A. Rodríguez-Iglesias N. Márquez-Ropero M. . (2020). Microglia actively remodel adult hippocampal neurogenesis through the phagocytosis secretome. J. Neurosci. 40, 1453–1482. doi: 10.1523/JNEUROSCI.0993-19.2019, 31896673 PMC7044727

[ref52] Dimitrakopoulos D. Kakogiannis D. Kazanis I. (2022). Heterogeneity of quiescent and active neural stem cells in the postnatal brain. Int. J. Dev. Biol. 66, 51–58. doi: 10.1387/ijdb.220010ik, 35238392

[ref53] Dong J. Pan Y.-B. Wu X.-R. He L.-N. Liu X.-D. Feng D.-F. . (2019). A neuronal molecular switch through cell-cell contact that regulates quiescent neural stem cells. Sci. Adv. 5:eaav4416. doi: 10.1126/sciadv.aav4416 30820459 PMC6392779

[ref54] Durakoglugil M. S. (2025). Reelin signaling as a translational rheostat: linking synaptic homeostasis to neurodevelopment and neurodegeneration. Front. Mol. Neurosci. 18:1731914. doi: 10.3389/fnmol.2025.1731914, 41416042 PMC12708547

[ref55] Edri R. Yaffe Y. Ziller M. J. Mutukula N. Volkman R. David E. . (2015). Analysing human neural stem cell ontogeny by consecutive isolation of notch active neural progenitors. Nat. Commun. 6:6500. doi: 10.1038/ncomms7500, 25799239 PMC4383005

[ref56] Ehret F. Moreno Traspas R. Neumuth M.-T. Hamann B. Lasse D. Kempermann G. (2021). Notch3-dependent effects on adult neurogenesis and hippocampus-dependent learning in a modified transgenic model of CADASIL. Front. Aging Neurosci. 13:617733. doi: 10.3389/fnagi.2021.617733, 34093162 PMC8177050

[ref57] Ekonomou A. Johnson M. Perry R. H. Perry E. K. Kalaria R. N. Minger S. L. . (2012). Increased neural progenitors in individuals with cerebral small vessel disease. Neuropathol. Appl. Neurobiol. 38, 344–353. doi: 10.1111/j.1365-2990.2011.01224.x, 21988073

[ref58] Elliott T. Liu K. Y. Hazan J. Wilson J. Vallipuram H. Jones K. . (2025). Hippocampal neurogenesis in adult primates: a systematic review. Mol. Psychiatry 30, 1195–1206. doi: 10.1038/s41380-024-02815-y, 39558003

[ref59] Erginousakis I. Papatheodoropoulos C. (2025). Modifying factors of adult hippocampal neurogenesis: a dorsoventral perspective in health and disease. Cells 15:59. doi: 10.3390/cells15010059, 41511343 PMC12786199

[ref60] Fame R. M. Kalugin P. N. Petrova B. Xu H. Soden P. A. Shipley F. B. . (2023). Defining diurnal fluctuations in mouse choroid plexus and CSF at high molecular, spatial, and temporal resolution. Nat. Commun. 14:3720. doi: 10.1038/s41467-023-39326-3, 37349305 PMC10287727

[ref61] Farkas E. Institóris Á. Domoki F. Mihály A. Bari F. (2006). The effect of pre- and posttreatment with diazoxide on the early phase of chronic cerebral hypoperfusion in the rat. Brain Res. 1087, 168–174. doi: 10.1016/j.brainres.2006.02.134, 16624259

[ref62] Fawcett J. W. Oohashi T. Pizzorusso T. (2019). The roles of perineuronal nets and the perinodal extracellular matrix in neuronal function. Nat. Rev. Neurosci. 20, 451–465. doi: 10.1038/s41583-019-0196-3, 31263252

[ref63] Ferreira A. C. Hemmer B. M. Philippi S. M. Grau-Perales A. B. Rosenstadt J. L. Liu H. . (2023). Neuronal TIMP2 regulates hippocampus-dependent plasticity and extracellular matrix complexity. Mol. Psychiatry 28, 3943–3954. doi: 10.1038/s41380-023-02296-5, 37914840 PMC10730400

[ref64] Fraga E. Medina V. Cuartero M. I. García-Culebras A. Bravo-Ferrer I. Hernández-Jiménez M. . (2023). Defective hippocampal neurogenesis underlies cognitive impairment by carotid stenosis-induced cerebral hypoperfusion in mice. Front. Cell. Neurosci. 17:1219847. doi: 10.3389/fncel.2023.1219847, 37636586 PMC10457159

[ref65] Fu C.-H. Iascone D. M. Petrof I. Hazra A. Zhang X. Pyfer M. S. . (2019). Early seizure activity accelerates depletion of hippocampal neural stem cells and impairs spatial discrimination in an Alzheimer’s disease model. Cell Rep. 27, 3741–3751.e4. doi: 10.1016/j.celrep.2019.05.101, 31242408 PMC6697001

[ref66] Fuentealba L. C. Obernier K. Alvarez-Buylla A. (2012). Adult neural stem cells bridge their niche. Cell Stem Cell 10, 698–708. doi: 10.1016/j.stem.2012.05.012, 22704510 PMC3726005

[ref67] Gallucci L. Sperber C. Guggisberg A. G. Kaller C. P. Heldner M. R. Monsch A. U. . (2024). Post-stroke cognitive impairment remains highly prevalent and disabling despite state-of-the-art stroke treatment. Int. J. Stroke 19, 888–897. doi: 10.1177/17474930241238637, 38425239

[ref68] Garone C. De Giorgio F. Carli S. (2024). Mitochondrial metabolism in neural stem cells and implications for neurodevelopmental and neurodegenerative diseases. J. Transl. Med. 22:238. doi: 10.1186/s12967-024-05041-w, 38438847 PMC10910780

[ref69] Gemma C. Bachstetter A. D. (2013). The role of microglia in adult hippocampal neurogenesis. Front. Cell. Neurosci. 7:229. doi: 10.3389/fncel.2013.00229, 24319411 PMC3837350

[ref70] Gileadi O. (2014). Structures of soluble guanylate cyclase: implications for regulatory mechanisms and drug development. Biochem. Soc. Trans. 42, 108–113. doi: 10.1042/BST20130228, 24450636 PMC3901396

[ref71] Godeanu S. Cătălin B. (2025). The complementary role of morphology in understanding microglial functional heterogeneity. Int. J. Mol. Sci. 26:3811. doi: 10.3390/ijms26083811, 40332469 PMC12027755

[ref72] Gómez-Gaviro M. V. Lovell-Badge R. Fernández-Avilés F. Lara-Pezzi E. (2012). The vascular stem cell niche. J. Cardiovasc. Transl. Res. 5, 618–630. doi: 10.1007/s12265-012-9371-x, 22644724

[ref73] Gonçalves J. T. Schafer S. T. Gage F. H. (2016). Adult neurogenesis in the hippocampus: from stem cells to behavior. Cell 167, 897–914. doi: 10.1016/j.cell.2016.10.021, 27814520

[ref74] Gonzalez-Reyes L. E. Chiang C.-C. Zhang M. Johnson J. Arrillaga-Tamez M. Couturier N. H. . (2019). Sonic hedgehog is expressed by hilar mossy cells and regulates cellular survival and neurogenesis in the adult hippocampus. Sci. Rep. 9:17402. doi: 10.1038/s41598-019-53192-4, 31758070 PMC6874678

[ref75] Haack F. Lemcke H. Ewald R. Rharass T. Uhrmacher A. M. (2015). Spatio-temporal model of endogenous ROS and raft-dependent WNT/β-catenin signaling driving cell fate commitment in human neural progenitor cells. PLoS Comput. Biol. 11:e1004106. doi: 10.1371/journal.pcbi.1004106 25793621 PMC4368204

[ref76] Hase Y. Jobson D. Cheong J. Gotama K. Maffei L. Hase M. . (2024). Hippocampal capillary pericytes in post-stroke and vascular dementias and Alzheimer’s disease and experimental chronic cerebral hypoperfusion. Acta Neuropathol. Commun. 12:29. doi: 10.1186/s40478-024-01737-8, 38360798 PMC10870440

[ref77] Hijazi S. Smit A. B. van Kesteren R. E. (2023). Fast-spiking parvalbumin-positive interneurons in brain physiology and Alzheimer’s disease. Mol. Psychiatry 28, 4954–4967. doi: 10.1038/s41380-023-02168-y, 37419975 PMC11041664

[ref78] Ioannidis K. Angelopoulos I. Gakis G. Karantzelis N. Spyroulias G. A. Lygerou Z. . (2021). 3D reconstitution of the neural stem cell niche: connecting the dots. Front. Bioeng. Biotechnol. 9:705470. doi: 10.3389/fbioe.2021.705470, 34778223 PMC8581349

[ref79] Iyer A. K. V. Tran K. T. Borysenko C. W. Cascio M. Camacho C. J. Blair H. C. . (2007). Tenascin cytotactin epidermal growth factor-like repeat binds epidermal growth factor receptor with low affinity. J. Cell. Physiol. 211, 748–758. doi: 10.1002/jcp.20986, 17311283

[ref80] Jang M.-H. Bonaguidi M. A. Kitabatake Y. Sun J. Song J. Kang E. . (2013). Secreted frizzled-related protein 3 regulates activity-dependent adult hippocampal neurogenesis. Cell Stem Cell 12, 215–223. doi: 10.1016/j.stem.2012.11.021, 23395446 PMC3569732

[ref81] Jin K. Minami M. Lan J. Q. Mao X. O. Batteur S. Simon R. P. . (2001). Neurogenesis in dentate subgranular zone and rostral subventricular zone after focal cerebral ischemia in the rat. Proc. Natl. Acad. Sci. USA 98, 4710–4715. doi: 10.1073/pnas.081011098, 11296300 PMC31899

[ref82] Jinno S. (2011). Topographic differences in adult neurogenesis in the mouse hippocampus: a stereology-based study using endogenous markers. Hippocampus 21, 467–480. doi: 10.1002/hipo.20762, 20087889

[ref83] Kadam S. D. Mulholland J. D. McDonald J. W. Comi A. M. (2009). Poststroke subgranular and rostral subventricular zone proliferation in a mouse model of neonatal stroke. J. Neurosci. Res. 87, 2653–2666. doi: 10.1002/jnr.22109, 19396874 PMC3654407

[ref84] Kalamakis G. Brüne D. Ravichandran S. Bolz J. Fan W. Ziebell F. . (2019). Quiescence modulates stem cell maintenance and regenerative capacity in the aging brain. Cell 176, 1407–1419.e14. doi: 10.1016/j.cell.2019.01.040, 30827680

[ref85] Katsimpardi L. Litterman N. K. Schein P. A. Miller C. M. Loffredo F. S. Wojtkiewicz G. R. . (2014). Vascular and neurogenic rejuvenation of the aging mouse brain by young systemic factors. Science 344, 630–634. doi: 10.1126/science.1251141, 24797482 PMC4123747

[ref86] Kee N. J. Preston E. Wojtowicz J. M. (2001). Enhanced neurogenesis after transient global ischemia in the dentate gyrus of the rat. Exp. Brain Res. 136, 313–320. doi: 10.1007/s002210000591, 11243473

[ref87] Kerever A. Schnack J. Vellinga D. Ichikawa N. Moon C. Arikawa-Hirasawa E. . (2007). Novel extracellular matrix structures in the neural stem cell niche capture the neurogenic factor fibroblast growth factor 2 from the extracellular milieu. Stem Cells 25, 2146–2157. doi: 10.1634/stemcells.2007-0082, 17569787

[ref88] Kernie S. G. Parent J. M. (2010). Forebrain neurogenesis after focal ischemic and traumatic brain injury. Neurobiol. Dis. 37, 267–274. doi: 10.1016/j.nbd.2009.11.002, 19909815 PMC2864918

[ref89] Keung A. J. de Juan-Pardo E. M. Schaffer D. V. Kumar S. (2011). Rho GTPases mediate the mechanosensitive lineage commitment of neural stem cells. Stem Cells 29, 1886–1897. doi: 10.1002/stem.746, 21956892 PMC5990277

[ref90] Kim T. A. Chen L. Ge S. (2021). The interplay of neurovasculature and adult hippocampal neurogenesis. Neurosci. Lett. 760:136071. doi: 10.1016/j.neulet.2021.136071, 34147540 PMC8683249

[ref91] Kim S.-H. Turnbull J. Guimond S. (2011). Extracellular matrix and cell signalling: the dynamic cooperation of integrin, proteoglycan and growth factor receptor. J. Endocrinol. 209, 139–151. doi: 10.1530/JOE-10-0377, 21307119

[ref92] Kirschen G. W. Kéry R. Ge S. (2018). The hippocampal neuro-glio-vascular network: metabolic vulnerability and potential neurogenic regeneration in disease. Brain Plasticity 3, 129–144. doi: 10.3233/BPL-170055, 30151338 PMC6091038

[ref93] Knopman D. S. Amieva H. Petersen R. C. Chételat G. Holtzman D. M. Hyman B. T. . (2021). Alzheimer disease. Nat. Rev. Dis. Primers 7:33. doi: 10.1038/s41572-021-00269-y, 33986301 PMC8574196

[ref94] Knoth R. Singec I. Ditter M. Pantazis G. Capetian P. Meyer R. P. . (2010). Murine features of neurogenesis in the human hippocampus across the lifespan from 0 to 100 years. PLoS One 5:e8809. doi: 10.1371/journal.pone.0008809, 20126454 PMC2813284

[ref95] Kohman R. A. DeYoung E. K. Bhattacharya T. K. Peterson L. N. Rhodes J. S. (2012). Wheel running attenuates microglia proliferation and increases expression of a proneurogenic phenotype in the hippocampus of aged mice. Brain Behav. Immun. 26, 803–810. doi: 10.1016/j.bbi.2011.10.006, 22056294 PMC3275652

[ref96] Kohwi M. Petryniak M. A. Long J. E. Ekker M. Obata K. Yanagawa Y. . (2007). A subpopulation of olfactory bulb GABAergic interneurons is derived from Emx1- and Dlx5/6-expressing progenitors. J. Neurosci. 27, 6878–6891. doi: 10.1523/JNEUROSCI.0254-07.2007, 17596436 PMC4917362

[ref97] Kosaka K. Mimura J. Itoh K. Satoh T. Shimojo Y. Kitajima C. . (2010). Role of Nrf2 and p62/ZIP in the neurite outgrowth by carnosic acid in PC12h cells. J. Biochem. 147, 73–81. doi: 10.1093/jb/mvp149, 19762340

[ref98] Kramer D. R. Fujii T. Ohiorhenuan I. Liu C. Y. (2016). Cortical spreading depolarization: pathophysiology, implications, and future directions. J. Clin. Neurosci. 24, 22–27. doi: 10.1016/j.jocn.2015.08.004, 26461911

[ref99] Kreisel T. Wolf B. Keshet E. Licht T. (2019). Unique role for dentate gyrus microglia in neuroblast survival and in VEGF-induced activation. Glia 67, 594–618. doi: 10.1002/glia.23505, 30453385

[ref100] Li G. Fang L. Fernández G. Pleasure S. J. (2013). The ventral hippocampus is the embryonic origin for adult neural stem cells in the dentate gyrus. Neuron 78, 658–672. doi: 10.1016/j.neuron.2013.03.019, 23643936 PMC3669230

[ref101] Li Q. Ford M. C. Lavik E. B. Madri J. A. (2006). Modeling the neurovascular niche: VEGF- and BDNF-mediated cross-talk between neural stem cells and endothelial cells: an *in vitro* study. J. Neurosci. Res. 84, 1656–1668. doi: 10.1002/jnr.21087, 17061253

[ref102] Li Z. Liang Z. Qi H. Luo X. Wang M. Du Z. . (2025). Lactate shuttling links histone lactylation to adult hippocampal neurogenesis in mice. Dev. Cell 60, 1182–1198.e8. doi: 10.1016/j.devcel.2024.12.021, 39765233

[ref103] Li Z.-Y. Yang X. Wang J.-K. Yan X.-X. Liu F. Zuo Y.-C. (2024). MFGE8 promotes adult hippocampal neurogenesis in rats following experimental subarachnoid hemorrhage via modifying the integrin β3/Akt signaling pathway. Cell Death Discovery 10:359. doi: 10.1038/s41420-024-02132-x, 39128910 PMC11317487

[ref104] Li Q. Zhang Z. Li Z. Zhou M. Liu B. Pan L. . (2013). ADAM17 is critical for multipolar exit and radial migration of neuronal intermediate progenitor cells in mice cerebral cortex. PLoS One 8:e65703. doi: 10.1371/journal.pone.0065703, 23755270 PMC3670835

[ref105] Liang Z. Jin N. Guo W. (2025). Neural stem cell heterogeneity in adult hippocampus. Cell Regeneration 14:6. doi: 10.1186/s13619-025-00222-4, 40053275 PMC11889326

[ref106] Lim D. A. Alvarez-Buylla A. (2016). The adult ventricular-subventricular zone (V-SVZ) and olfactory bulb (OB) neurogenesis. Cold Spring Harb. Perspect. Biol. 8:a018820. doi: 10.1101/cshperspect.a018820, 27048191 PMC4852803

[ref107] Lin R. Cai J. Kenyon L. Iozzo R. Rosenwasser R. Iacovitti L. (2019). Systemic factors trigger vasculature cells to drive notch signaling and neurogenesis in neural stem cells in the adult brain. Stem Cells 37, 395–406. doi: 10.1002/stem.2947, 30431198 PMC7028145

[ref108] Lin R. Lang M. Heinsinger N. Stricsek G. Zhang J. Iozzo R. . (2018). Stepwise impairment of neural stem cell proliferation and neurogenesis concomitant with disruption of blood-brain barrier in recurrent ischemic stroke. Neurobiol. Dis. 115, 49–58. doi: 10.1016/j.nbd.2018.03.013, 29605425

[ref109] Lin J. H.-C. Takano T. Arcuino G. Wang X. Hu F. Darzynkiewicz Z. . (2007). Purinergic signaling regulates neural progenitor cell expansion and neurogenesis. Dev. Biol. 302, 356–366. doi: 10.1016/j.ydbio.2006.09.017, 17188262 PMC1924912

[ref110] Lippmann K. (2024). A reduction in the readily releasable vesicle pool impairs GABAergic inhibition in the hippocampus after blood-brain barrier dysfunction. Int. J. Mol. Sci. 25:6862. doi: 10.3390/ijms25136862, 38999971 PMC11241665

[ref111] Liu Y. Bilen M. McNicoll M.-M. Harris R. A. Fong B. C. Iqbal M. A. . (2023). Early postnatal defects in neurogenesis in the 3xTg mouse model of Alzheimer’s disease. Cell Death Dis. 14:138. doi: 10.1038/s41419-023-05650-1, 36801910 PMC9938901

[ref112] Llorente V. Velarde P. Desco M. Gómez-Gaviro M. V. (2022). Current understanding of the neural stem cell niches. Cells 11:3002. doi: 10.3390/cells11193002, 36230964 PMC9563325

[ref113] Long K. R. Huttner W. B. (2019). How the extracellular matrix shapes neural development. Open Biol. 9:180216. doi: 10.1098/rsob.180216, 30958121 PMC6367132

[ref114] López-Juárez A. Howard J. Ullom K. Howard L. Grande A. Pardo A. . (2013). Gsx2 controls region-specific activation of neural stem cells and injury-induced neurogenesis in the adult subventricular zone. Genes Dev. 27, 1272–1287. doi: 10.1101/gad.217539.113, 23723414 PMC3690400

[ref115] Lugert S. Basak O. Knuckles P. Haussler U. Fabel K. Götz M. . (2010). Quiescent and active hippocampal neural stem cells with distinct morphologies respond selectively to physiological and pathological stimuli and aging. Cell Stem Cell 6, 445–456. doi: 10.1016/j.stem.2010.03.017, 20452319

[ref116] Luque T. Kang M. S. Schaffer D. V. Kumar S. (2016). Microelastic mapping of the rat dentate gyrus. R. Soc. Open Sci. 3:150702. doi: 10.1098/rsos.150702, 27152213 PMC4852636

[ref117] Ma D. K. Jang M.-H. Guo J. U. Kitabatake Y. Chang M.-L. Pow-Anpongkul N. . (2009). Neuronal activity-induced Gadd45b promotes epigenetic DNA demethylation and adult neurogenesis. Science 323, 1074–1077. doi: 10.1126/science.1166859, 19119186 PMC2726986

[ref118] Ma L. Wei Q. Jiang M. Wu Y. Liu X. Yang Q. . (2025). Hippocampal neurogenesis in Alzheimer’s disease: multimodal therapeutics and the neurogenic impairment index framework. Int. J. Mol. Sci. 26:6105. doi: 10.3390/ijms26136105, 40649882 PMC12250571

[ref119] Mäkelä J. Koivuniemi R. Korhonen L. Lindholm D. (2010). Interferon-gamma produced by microglia and the neuropeptide PACAP have opposite effects on the viability of neural progenitor cells. PLoS One 5:e11091. doi: 10.1371/journal.pone.0011091, 20559421 PMC2885411

[ref120] Marathe S. Jaquet M. Annoni J.-M. Alberi L. (2017). Jagged1 is altered in Alzheimer’s disease and regulates spatial memory processing. Front. Cell. Neurosci. 11:220. doi: 10.3389/fncel.2017.00220, 28848392 PMC5552758

[ref121] McAvoy K. M. Scobie K. N. Berger S. Russo C. Guo N. Decharatanachart P. . (2016). Modulating neuronal competition dynamics in the dentate gyrus to rejuvenate aging memory circuits. Neuron 91, 1356–1373. doi: 10.1016/j.neuron.2016.08.009, 27593178 PMC5033725

[ref122] Mehta A. Prabhakar M. Kumar P. Deshmukh R. Sharma P. L. (2013). Excitotoxicity: bridge to various triggers in neurodegenerative disorders. Eur. J. Pharmacol. 698, 6–18. doi: 10.1016/j.ejphar.2012.10.032, 23123057

[ref123] Melani A. Cipriani S. Corti F. Pedata F. (2010). Effect of intravenous administration of dipyridamole in a rat model of chronic cerebral ischemia. Ann. N. Y. Acad. Sci. 1207, 89–96. doi: 10.1111/j.1749-6632.2010.05732.x, 20955431

[ref124] Mercier F. (2016). Fractones: extracellular matrix niche controlling stem cell fate and growth factor activity in the brain in health and disease. Cell. Mol. Life Sci. 73, 4661–4674. doi: 10.1007/s00018-016-2314-y, 27475964 PMC11108427

[ref125] Miller S. M. Sahay A. (2019). Functions of adult-born neurons in hippocampal memory interference and indexing. Nat. Neurosci. 22, 1565–1575. doi: 10.1038/s41593-019-0484-2, 31477897 PMC7397477

[ref126] Ming G.-L. Song H. (2011). Adult neurogenesis in the mammalian brain: significant answers and significant questions. Neuron 70, 687–702. doi: 10.1016/j.neuron.2011.05.001, 21609825 PMC3106107

[ref127] Mishra S. K. Braun N. Shukla V. Füllgrabe M. Schomerus C. Korf H.-W. . (2006). Extracellular nucleotide signaling in adult neural stem cells: synergism with growth factor-mediated cellular proliferation. Development 133, 675–684. doi: 10.1242/dev.02233, 16436623

[ref128] Mizrak D. Levitin H. M. Delgado A. C. Crotet V. Yuan J. Chaker Z. . (2019). Single-cell analysis of regional differences in adult V-SVZ neural stem cell lineages. Cell Rep. 26, 394–406.e5. doi: 10.1016/j.celrep.2018.12.044, 30625322 PMC6368857

[ref129] Monje M. L. Toda H. Palmer T. D. (2003). Inflammatory blockade restores adult hippocampal neurogenesis. Science 302, 1760–1765. doi: 10.1126/science.1088417, 14615545

[ref130] Moreno-Jiménez E. P. Flor-García M. Terreros-Roncal J. Rábano A. Cafini F. Pallas-Bazarra N. . (2019). Adult hippocampal neurogenesis is abundant in neurologically healthy subjects and drops sharply in patients with Alzheimer’s disease. Nat. Med. 25, 554–560. doi: 10.1038/s41591-019-0375-9, 30911133

[ref131] Moreno-Jiménez E. P. Terreros-Roncal J. Flor-García M. Rábano A. Llorens-Martín M. (2021). Evidences for adult hippocampal neurogenesis in humans. J. Neurosci. 41, 2541–2553. doi: 10.1523/JNEUROSCI.0675-20.2020, 33762406 PMC8018741

[ref132] Moser M. B. Moser E. I. (1998). Functional differentiation in the hippocampus. Hippocampus 8, 608–619. doi: 10.1002/(SICI)1098-1063(1998)8:6<608::AID-HIPO3>3.0.CO;2-7, 9882018

[ref133] Muñoz-Espín D. Serrano M. (2014). Cellular senescence: from physiology to pathology. Nat. Rev. Mol. Cell Biol. 15, 482–496. doi: 10.1038/nrm3823, 24954210

[ref134] Murillo-Carretero M. Torroglosa A. Castro C. Villalobo A. Estrada C. (2009). S-nitrosylation of the epidermal growth factor receptor: a regulatory mechanism of receptor tyrosine kinase activity. Free Radic. Biol. Med. 46, 471–479. doi: 10.1016/j.freeradbiomed.2008.10.048, 19056486

[ref135] Nakajo H. Cao R. Mula S. A. McKetney J. Silva N. J. Li K. H. . (2026). Extracellular matrix proteolysis maintains synapse plasticity during brain development. Nat. Neurosci. 29, 567–580. doi: 10.1038/s41593-025-02153-4, 41430471 PMC12971489

[ref136] Nimmerjahn A. Kirchhoff F. Helmchen F. (2005). Resting microglial cells are highly dynamic surveillants of brain parenchyma in vivo. Science 308, 1314–1318. doi: 10.1126/science.1110647, 15831717

[ref137] Niv F. Keiner S. Krishna K. Witte O. W. Lie D. C. Redecker C. (2012). Aberrant neurogenesis after stroke: a retroviral cell labeling study. Stroke 43, 2468–2475. doi: 10.1161/STROKEAHA.112.660977, 22738919

[ref138] Noguchi H. Arela J. C. Ngo T. Cocas L. Pleasure S. (2023). Shh from mossy cells contributes to preventing NSC pool depletion after seizure-induced neurogenesis and in aging. eLife 12:RP91263. doi: 10.7554/eLife.91263, 38079471 PMC10712957

[ref139] Noguera-Troise I. Daly C. Papadopoulos N. J. Coetzee S. Boland P. Gale N. W. . (2006). Blockade of Dll4 inhibits tumour growth by promoting non-productive angiogenesis. Nature 444, 1032–1037. doi: 10.1038/nature05355, 17183313

[ref140] Ohtaki H. Fujimoto T. Sato T. Kishimoto K. Fujimoto M. Moriya M. . (2006). Progressive expression of vascular endothelial growth factor (VEGF) and angiogenesis after chronic ischemic hypoperfusion in rat. Acta Neurochir. Suppl. 96, 283–287. doi: 10.1007/3-211-30714-1_61, 16671472

[ref141] Otsuki L. Brand A. H. (2017). The vasculature as a neural stem cell niche. Neurobiol. Dis. 107, 4–14. doi: 10.1016/j.nbd.2017.01.010, 28132930

[ref142] Ottone C. Krusche B. Whitby A. Clements M. Quadrato G. Pitulescu M. E. . (2014). Direct cell-cell contact with the vascular niche maintains quiescent neural stem cells. Nat. Cell Biol. 16, 1045–1056. doi: 10.1038/ncb3045, 25283993 PMC4298702

[ref143] Packer M. A. Stasiv Y. Benraiss A. Chmielnicki E. Grinberg A. Westphal H. . (2003). Nitric oxide negatively regulates mammalian adult neurogenesis. Proc. Natl. Acad. Sci. USA 100, 9566–9571. doi: 10.1073/pnas.1633579100, 12886012 PMC170958

[ref144] Paolicelli R. C. Bolasco G. Pagani F. Maggi L. Scianni M. Panzanelli P. . (2011). Synaptic pruning by microglia is necessary for normal brain development. Science 333, 1456–1458. doi: 10.1126/science.1202529, 21778362

[ref145] Paris I. Savage J. C. Escobar L. Abiega O. Gagnon S. Hui C.-W. . (2018). ProMoIJ: a new tool for automatic three-dimensional analysis of microglial process motility. Glia 66, 828–845. doi: 10.1002/glia.23287, 29288586

[ref146] Park C. Kang M. Kwon Y. K. Chung J. H. Ahn H. Huh Y. (2001). Inhibition of neuronal nitric oxide synthase enhances cell proliferation in the dentate gyrus of the adrenalectomized rat. Neurosci. Lett. 309, 9–12. doi: 10.1016/S0304-3940(01)02003-1, 11489534

[ref147] Pathak M. M. Nourse J. L. Tran T. Hwe J. Arulmoli J. Le D. T. . (2014). Stretch-activated ion channel Piezo1 directs lineage choice in human neural stem cells. Proc. Natl. Acad. Sci. USA 111, 16148–16153. doi: 10.1073/pnas.1409802111, 25349416 PMC4234578

[ref148] Pilz G.-A. Bottes S. Betizeau M. Jörg D. J. Carta S. April S. . (2018). Live imaging of neurogenesis in the adult mouse hippocampus. Science 359, 658–662. doi: 10.1126/science.aao5056, 29439238 PMC6986926

[ref149] Porcheri C. Suter U. Jessberger S. (2014). Dissecting integrin-dependent regulation of neural stem cell proliferation in the adult brain. J. Neurosci. 34, 5222–5232. doi: 10.1523/JNEUROSCI.4928-13.2014, 24719101 PMC6608998

[ref150] Qian D. Dong Y. Liu X. Chen Y. Ge H. Liu J. . (2024). Salidroside promotes the repair of spinal cord injury by inhibiting astrocyte polarization, promoting neural stem cell proliferation and neuronal differentiation. Cell Death Discov. 10:224. doi: 10.1038/s41420-024-01989-2 38724500 PMC11082153

[ref151] Qin D. Liu Q. Liang Z. Cai H. Luo X. Wang M. . (2026). Adult neural stem cells mediate hippocampal synapse elimination for circuit homeostasis through MERTK. Proc. Natl. Acad. Sci. USA 123:e2517096123. doi: 10.1073/pnas.2517096123, 41499403 PMC12799105

[ref152] Radakovits R. Barros C. S. Belvindrah R. Patton B. Müller U. (2009). Regulation of radial glial survival by signals from the meninges. J. Neurosci. 29, 7694–7705. doi: 10.1523/JNEUROSCI.5537-08.2009, 19535581 PMC2738639

[ref153] Radosinska D. Radosinska J. (2025). The link between matrix metalloproteinases and Alzheimer’s disease pathophysiology. Mol. Neurobiol. 62, 885–899. doi: 10.1007/s12035-024-04315-0, 38935232 PMC11711632

[ref154] Rammensee S. Kang M. S. Georgiou K. Kumar S. Schaffer D. V. (2017). Dynamics of mechanosensitive neural stem cell differentiation. Stem Cells 35, 497–506. doi: 10.1002/stem.2489, 27573749 PMC5285406

[ref155] Ribak C. E. Shapiro L. A. Perez Z. D. Spigelman I. (2009). Microglia-associated granule cell death in the normal adult dentate gyrus. Brain Struct. Funct. 214, 25–35. doi: 10.1007/s00429-009-0231-7, 19936784 PMC2782120

[ref156] Ridgway J. Zhang G. Wu Y. Stawicki S. Liang W.-C. Chanthery Y. . (2006). Inhibition of Dll4 signalling inhibits tumour growth by deregulating angiogenesis. Nature 444, 1083–1087. doi: 10.1038/nature05313, 17183323

[ref157] Ruan L. Wang B. ZhuGe Q. Jin K. (2015). Coupling of neurogenesis and angiogenesis after ischemic stroke. Brain Res. 1623, 166–173. doi: 10.1016/j.brainres.2015.02.042, 25736182 PMC4552615

[ref158] Ryu J. R. Hong C. J. Kim J. Y. Kim E.-K. Sun W. Yu S.-W. (2016). Control of adult neurogenesis by programmed cell death in the mammalian brain. Mol. Brain 9:43. doi: 10.1186/s13041-016-0224-4, 27098178 PMC4839132

[ref159] Ryu Y. Iwashita M. Lee W. Uchimura K. Kosodo Y. (2021). A shift in tissue stiffness during hippocampal maturation correlates to the pattern of neurogenesis and composition of the extracellular matrix. Front. Aging Neurosci. 13:709620. doi: 10.3389/fnagi.2021.709620, 34393762 PMC8361493

[ref160] Schaapsmeerders P. van Uden I. W. M. Tuladhar A. M. Maaijwee N. A. M. van Dijk E. J. Rutten-Jacobs L. C. A. . (2015). Ipsilateral hippocampal atrophy is associated with long-term memory dysfunction after ischemic stroke in young adults. Hum. Brain Mapp. 36, 2432–2442. doi: 10.1002/hbm.22782, 25757914 PMC6869088

[ref161] Schafer D. P. Lehrman E. K. Kautzman A. G. Koyama R. Mardinly A. R. Yamasaki R. . (2012). Microglia sculpt postnatal neural circuits in an activity and complement-dependent manner. Neuron 74, 691–705. doi: 10.1016/j.neuron.2012.03.026, 22632727 PMC3528177

[ref162] Scharfman H. E. Myers C. E. (2013). Hilar mossy cells of the dentate gyrus: a historical perspective. Front. Neural Circuits 6:106. doi: 10.3389/fncir.2012.00106, 23420672 PMC3572871

[ref163] Schmitt O. Eipert P. Wang Y. Kanoke A. Rabiller G. Liu J. (2024). Connectome-based prediction of functional impairment in experimental stroke models. PLoS One 19:e0310743. doi: 10.1371/journal.pone.0310743, 39700116 PMC11658581

[ref164] Sharp F. R. Liu J. Bernabeu R. (2002). Neurogenesis following brain ischemia. Dev. Brain Res. 134, 23–30. doi: 10.1016/S0165-3806(01)00286-3, 11947934

[ref165] Shen J. Wang D. Wang X. Gupta S. Ayloo B. Wu S. . (2019). Neurovascular coupling in the dentate gyrus regulates adult hippocampal neurogenesis. Neuron 103, 878–890.e3. doi: 10.1016/j.neuron.2019.05.045, 31257104 PMC6728189

[ref166] Shibata M. Ohtani R. Ihara M. Tomimoto H. (2004). White matter lesions and glial activation in a novel mouse model of chronic cerebral hypoperfusion. Stroke 35, 2598–2603. doi: 10.1161/01.STR.0000143725.19053.60, 15472111

[ref167] Siddiqui T. Cosacak M. I. Popova S. Bhattarai P. Yilmaz E. Lee A. J. . (2023). Nerve growth factor receptor (Ngfr) induces neurogenic plasticity by suppressing reactive astroglial Lcn2/Slc22a17 signaling in Alzheimer’s disease. npj Regener. Med. 8:33. doi: 10.1038/s41536-023-00311-5, 37429840 PMC10333226

[ref168] Sierra A. Encinas J. M. Deudero J. J. P. Chancey J. H. Enikolopov G. Overstreet-Wadiche L. S. . (2010). Microglia shape adult hippocampal neurogenesis through apoptosis-coupled phagocytosis. Cell Stem Cell 7, 483–495. doi: 10.1016/j.stem.2010.08.014, 20887954 PMC4008496

[ref169] Silva-Vargas V. Maldonado-Soto A. R. Mizrak D. Codega P. Doetsch F. (2016). Age-dependent niche signals from the choroid plexus regulate adult neural stem cells. Cell Stem Cell 19, 643–652. doi: 10.1016/j.stem.2016.06.013, 27452173

[ref170] Solano Fonseca R. Mahesula S. Apple D. M. Raghunathan R. Dugan A. Cardona A. . (2016). Neurogenic niche microglia undergo positional remodeling and progressive activation contributing to age-associated reductions in neurogenesis. Stem Cells Dev. 25, 542–555. doi: 10.1089/scd.2015.0319, 26857912 PMC4817564

[ref171] Song J. Zhong C. Bonaguidi M. A. Sun G. J. Hsu D. Gu Y. . (2012). Neuronal circuitry mechanism regulating adult quiescent neural stem-cell fate decision. Nature 489, 150–154. doi: 10.1038/nature11306, 22842902 PMC3438284

[ref172] Spalding K. L. Bergmann O. Alkass K. Bernard S. Salehpour M. Huttner H. B. . (2013). Dynamics of hippocampal neurogenesis in adult humans. Cell 153, 1219–1227. doi: 10.1016/j.cell.2013.05.002, 23746839 PMC4394608

[ref173] Strange B. A. Witter M. P. Lein E. S. Moser E. I. (2014). Functional organization of the hippocampal longitudinal axis. Nat. Rev. Neurosci. 15, 655–669. doi: 10.1038/nrn3785 25234264

[ref174] Sunatani Y. Sakasai R. Matsui T. Iwabuchi K. (2024). 53BP1 regulates the self-renewal ability of neural stem/progenitor cells through modulating mitochondrial homeostasis. Biochem. Biophys. Res. Commun. 734:150776. doi: 10.1016/j.bbrc.2024.150776, 39368367

[ref175] Sung P.-S. Lin P.-Y. Liu C.-H. Su H.-C. Tsai K.-J. (2020). Neuroinflammation and neurogenesis in Alzheimer’s disease and potential therapeutic approaches. Int. J. Mol. Sci. 21:701. doi: 10.3390/ijms21030701, 31973106 PMC7037892

[ref176] Tanaka R. Yamashiro K. Mochizuki H. Cho N. Onodera M. Mizuno Y. . (2004). Neurogenesis after transient global ischemia in the adult hippocampus visualized by improved retroviral vector. Stroke 35, 1454–1459. doi: 10.1161/01.STR.0000126480.40967.b3, 15073392

[ref177] Tian Z. Ji X. Liu J. (2022). Neuroinflammation in vascular cognitive impairment and dementia: current evidence, advances, and prospects. Int. J. Mol. Sci. 23:6224. doi: 10.3390/ijms23116224, 35682903 PMC9181710

[ref178] Tincer G. Mashkaryan V. Bhattarai P. Kizil C. (2016). Neural stem/progenitor cells in Alzheimer’s disease. Yale J. Biol. Med. 89, 23–35. 27505014 PMC4797833

[ref179] Tomita S. Ueno M. Sakamoto M. Kitahama Y. Ueki M. Maekawa N. . (2003). Defective brain development in mice lacking the Hif-1α gene in neural cells. Mol. Cell. Biol. 23, 6739–6749. doi: 10.1128/MCB.23.19.6739-6749.2003, 12972594 PMC193947

[ref180] Torrisi F. Denaro S. Ragonese J. D’Aprile S. Zappalà A. Parenti R. (2025). Neural stem cells fate under neuroinflammatory conditions and oxidative stress response. Front. Cell. Neurosci. 19:1736865. doi: 10.3389/fncel.2025.1736865, 41480493 PMC12753481

[ref181] Torroglosa A. Murillo-Carretero M. Romero-Grimaldi C. Matarredona E. R. Campos-Caro A. Estrada C. (2007). Nitric oxide decreases subventricular zone stem cell proliferation by inhibition of epidermal growth factor receptor and phosphoinositide-3-kinase/Akt pathway. Stem Cells 25, 88–97. doi: 10.1634/stemcells.2006-0131, 16960136

[ref182] Tozuka Y. Fukuda S. Namba T. Seki T. Hisatsune T. (2005). GABAergic excitation promotes neuronal differentiation in adult hippocampal progenitor cells. Neuron 47, 803–815. doi: 10.1016/j.neuron.2005.08.023, 16157276

[ref183] Trinchero M. F. Herrero M. Monzón-Salinas M. C. Schinder A. F. (2019). Experience-dependent structural plasticity of adult-born neurons in the aging hippocampus. Front. Neurosci. 13:739. doi: 10.3389/fnins.2019.00739, 31379489 PMC6651579

[ref184] Türeyen K. Vemuganti R. Sailor K. A. Dempsey R. J. (2004). Transient focal cerebral ischemia-induced neurogenesis in the dentate gyrus of the adult mouse. J. Neurosurg. 101, 799–805. doi: 10.3171/jns.2004.101.5.0799, 15540918

[ref185] van Deursen J. M. (2014). The role of senescent cells in ageing. Nature 509, 439–446. doi: 10.1038/nature13193, 24848057 PMC4214092

[ref186] Verkhratsky A. Matteoli M. Parpura V. Mothet J.-P. Zorec R. (2016). Astrocytes as secretory cells of the central nervous system: idiosyncrasies of vesicular secretion. EMBO J. 35, 239–257. doi: 10.15252/embj.201592705, 26758544 PMC4741299

[ref187] Vockert N. Perosa V. Ziegler G. Schreiber F. Priester A. Spallazzi M. . (2021). Hippocampal vascularization patterns exert local and distant effects on brain structure but not vascular pathology in old age. Brain Commun. 3:fcab127. doi: 10.1093/braincomms/fcab127, 34222874 PMC8249103

[ref188] Vogel J. W. La Joie R. Grothe M. J. Diaz-Papkovich A. Doyle A. Vachon-Presseau E. . (2020). A molecular gradient along the longitudinal axis of the human hippocampus informs large-scale behavioral systems. Nat. Commun. 11:960. doi: 10.1038/s41467-020-14518-3, 32075960 PMC7031290

[ref189] Vukovic J. Colditz M. J. Blackmore D. G. Ruitenberg M. J. Bartlett P. F. (2012). Microglia modulate hippocampal neural precursor activity in response to exercise and aging. J. Neurosci. 32, 6435–6443. doi: 10.1523/JNEUROSCI.5925-11.2012, 22573666 PMC6621117

[ref190] Walter J. Keiner S. Witte O. W. Redecker C. (2010). Differential stroke-induced proliferative response of distinct precursor cell subpopulations in the young and aged dentate gyrus. Neuroscience 169, 1279–1286. doi: 10.1016/j.neuroscience.2010.05.035, 20570606

[ref191] Wander C. M. Song J. (2021). The neurogenic niche in Alzheimer’s disease. Neurosci. Lett. 762:136109. doi: 10.1016/j.neulet.2021.136109, 34271133 PMC9013442

[ref192] Wang Z.-F. Fessler E. B. Chuang D.-M. (2011). Beneficial effects of mood stabilizers lithium, valproate and lamotrigine in experimental stroke models. Acta Pharmacol. Sin. 32, 1433–1445. doi: 10.1038/aps.2011.140, 22056617 PMC4010202

[ref193] Wang X. Harris R. E. Bayston L. J. Ashe H. L. (2008). Type IV collagens regulate BMP signalling in Drosophila. Nature 455, 72–77. doi: 10.1038/nature07214, 18701888

[ref194] Wang S. Pan Y. Zhang C. Zhao Y. Wang H. Ma H. . (2024). Transcriptome analysis reveals dynamic microglial-induced A1 astrocyte reactivity via C3/C3aR/NF-κB signaling after ischemic stroke. Mol. Neurobiol. 61, 10246–10270. doi: 10.1007/s12035-024-04210-8, 38713438

[ref195] Weinhard L. di Bartolomei G. Bolasco G. Machado P. Schieber N. L. Neniskyte U. . (2018). Microglia remodel synapses by presynaptic trogocytosis and spine head filopodia induction. Nat. Commun. 9:1228. doi: 10.1038/s41467-018-03566-5, 29581545 PMC5964317

[ref196] Wesley U. V. Hatcher J. F. Ayvaci E. R. Klemp A. Dempsey R. J. (2017). Regulation of dipeptidyl peptidase IV in the post-stroke rat brain and in vitro ischemia: implications for chemokine-mediated neural progenitor cell migration and angiogenesis. Mol. Neurobiol. 54, 4973–4985. doi: 10.1007/s12035-016-0039-4, 27525674 PMC5985827

[ref197] Wilhelmsson U. Faiz M. de Pablo Y. Sjöqvist M. Andersson D. Widestrand Å. . (2012). Astrocytes negatively regulate neurogenesis through the Jagged1-mediated notch pathway. Stem Cells 30, 2320–2329. doi: 10.1002/stem.1196, 22887872

[ref198] Willis C. M. Nicaise A. M. Krzak G. Ionescu R. B. Pappa V. D’Angelo A. . (2022). Soluble factors influencing the neural stem cell niche in brain physiology, inflammation, and aging. Exp. Neurol. 355:114124. doi: 10.1016/j.expneurol.2022.114124 35644426

[ref199] Yagita Y. Kitagawa K. Ohtsuki T. Takasawa K. Miyata T. Okano H. . (2001). Neurogenesis by progenitor cells in the ischemic adult rat hippocampus. Stroke 32, 1890–1896. doi: 10.1161/01.STR.32.8.1890, 11486122

[ref200] Yan Y.-P. Sailor K. A. Vemuganti R. Dempsey R. J. (2006). Insulin-like growth factor-1 is an endogenous mediator of focal ischemia-induced neural progenitor proliferation. Eur. J. Neurosci. 24, 45–54. doi: 10.1111/j.1460-9568.2006.04872.x, 16882007

[ref201] Yeh C.-Y. Asrican B. Moss J. Quintanilla L. J. He T. Mao X. . (2018). Mossy cells control adult neural stem cell quiescence and maintenance through a dynamic balance between direct and indirect pathways. Neuron 99, 493–510.e4. doi: 10.1016/j.neuron.2018.07.010, 30057205 PMC6092757

[ref202] Yi L. X. Zeng L. Wang Q. Tan E. K. Zhou Z. D. (2024). Reelin links Apolipoprotein E4, tau, and amyloid-β in Alzheimer’s disease. Ageing Res. Rev. 98:102339. doi: 10.1016/j.arr.2024.102339 38754634

[ref203] Yoshimura S. Takagi Y. Harada J. Teramoto T. Thomas S. S. Waeber C. . (2001). FGF-2 regulation of neurogenesis in adult hippocampus after brain injury. Proc. Natl. Acad. Sci. USA 98, 5874–5879. doi: 10.1073/pnas.101034998, 11320217 PMC33306

[ref204] Yoshimura S. Teramoto T. Whalen M. J. Irizarry M. C. Takagi Y. Qiu J. . (2003). FGF-2 regulates neurogenesis and degeneration in the dentate gyrus after traumatic brain injury in mice. J. Clin. Invest. 112, 1202–1210. doi: 10.1172/JCI200316618 14561705 PMC213483

[ref205] Zhang R. L. Chopp M. Roberts C. Liu X. Wei M. Nejad-Davarani S. P. . (2014). Stroke increases neural stem cells and angiogenesis in the neurogenic niche of the adult mouse. PLoS One 9:e113972. doi: 10.1371/journal.pone.0113972, 25437857 PMC4250076

[ref206] Zhang F. Fu Y. Jimenez-Cyrus D. Zhao T. Shen Y. Sun Y. . (2025). m6A/YTHDF2-mediated mRNA decay targets TGF-β signaling to suppress the quiescence acquisition of early postnatal mouse hippocampal NSCs. Cell Stem Cell 32, 144–156.e8. doi: 10.1016/j.stem.2024.10.002, 39476834 PMC11698649

[ref207] Zhao T. Pan P. Jia Y. Zhou Y. Zhang X. Guan H. . (2025). Crossing pathological boundaries: multi-target restoration of the neurovascular unit in Alzheimer’s and vascular dementia—from modern therapeutics to traditional Chinese medicine. Aging Dis. 17, 2353–2381. doi: 10.14336/AD.2025.0801, 40901978 PMC13437122

[ref208] Zhou Y. Bond A. M. Shade J. E. Zhu Y. Davis C. O. Wang X. . (2018). Autocrine Mfge8 signaling prevents developmental exhaustion of the adult neural stem cell pool. Cell Stem Cell 23, 444–452.e4. doi: 10.1016/j.stem.2018.08.005, 30174295 PMC6128767

[ref209] Zhu Y. Oganesian A. Keene D. R. Sandell L. J. (1999). Type IIA procollagen containing the cysteine-rich amino propeptide is deposited in the extracellular matrix of prechondrogenic tissue and binds to TGF-β1 and BMP-2. J. Cell Biol. 144, 1069–1080. doi: 10.1083/jcb.144.5.1069, 10085302 PMC2148200

[ref210] Zhuang J. Wei Q. Lin Z. Zhou C. (2015). Effects of ADAM10 deletion on Notch-1 signaling pathway and neuronal maintenance in adult mouse brain. Gene 555, 150–158. doi: 10.1016/j.gene.2014.10.056, 25445276

[ref211] Ziebell F. Dehler S. Martin-Villalba A. Marciniak-Czochra A. (2018). Revealing age-related changes of adult hippocampal neurogenesis using mathematical models. Development 145:dev153544. doi: 10.1242/dev.153544, 29229768 PMC5825879

